# Mechanism of Gating and Isoform-Specific Inhibition in Renal CLC Chloride Channels

**DOI:** 10.64898/2026.02.17.706469

**Published:** 2026-02-18

**Authors:** Chih-Ta Chien, Briana L. Sobecks, Alexander S. Powers, Jürgen Kreiter, Anindita Das, Chloe N. Barry, Muyuan Chen, Andrew Hinman, Camille F. Petrakian, Natasa Trifkovic, Brianna Williams, Chase A.P. Wood, Mengyuan Xu, Ron O. Dror, Wah Chiu, Merritt Maduke

**Affiliations:** 1 co-first authors; 2 Department of Bioengineering, Stanford University, Stanford, CA 94305; 3 current affiliation: National Institute of Diabetes and Digestive and Kidney Diseases (NIDDK), National Institutes of Health, Bethesda, MD, 20892; 4 Department of Molecular and Cellular Physiology, Stanford University School of Medicine, Stanford, CA 94305; 5 Department of Chemical Engineering, Stanford University, Stanford, CA 94305; 6 Department of Computer Science, Stanford University, Stanford, CA 94305; 7 Department of Structural Biology, Stanford University, Stanford, CA 94305; 8 Institute for Computational and Mathematical Engineering, Stanford University, Stanford, CA 94305; 9 Department of Chemistry, Stanford University, Stanford CA; 10 current affiliation: Institute for Medical Physics and Biophysics, University of Veterinary Medicine Vienna, 1210 Vienna, Austria; 11 Innovative Medicines Accelerator, Stanford University, Stanford, CA 94305; 12 Division of CryoEM and Bioimaging, SSRL, SLAC National Accelerator Laboratory, Stanford University, Menlo Park 94025; 13 Department of Microbiology and Immunology, Stanford University School of Medicine, Stanford, CA 94305

**Keywords:** CLC-Ka chloride channel, isoform selectivity, cryo-EM, molecular dynamics simulations, channel gating, Major: Biological Sciences, Minor: Biophysics and Computational Biology

## Abstract

Hyponatremia is a prevalent disorder marked by excess water retention and substantial morbidity, motivating interest in the CLC-Ka chloride channel as a therapeutic target. Selectively inhibiting CLC-Ka without affecting the closely related CLC-Kb is essential for preventing serious side effects. However, developing isoform-selective inhibitors has been challenging because most small molecules do not distinguish between CLC-Ka and CLC-Kb, and the basis for selectivity in the few known exceptions remains unclear. The small molecule BIM1 preferentially inhibits CLC-Ka over CLC-Kb, providing an opportunity to dissect isoform-specific pharmacology. To investigate this mechanism, we determined cryo-EM structures of BIM1 and BIM15, a related nonselective analog, bound to a CLC-K variant engineered to match the human CLC-Ka binding pocket. Structural and computational analyses reveal that inhibition and isoform selectivity are anchored by interactions with a conserved lysine, with surrounding binding-site residues subtly tuning the local electrostatic environment to promote or disfavor these contacts. These analyses further identify a dynamic extracellular loop that intermittently occludes the access pathway, indicating its role as a gate for ions and inhibitors. BIM15 engages this gating loop more extensively than BIM1, suggesting that differential loop engagement contributes to inhibitor selectivity. To probe how gating reshapes this region, we solved the structure in the presence of Ca^2+^, which favors channel opening, and found the gating loop ordered and withdrawn from the pathway. Together, these findings elucidate how CLC-K channels gate and how subtle binding-site differences and loop dynamics shape isoform-specific drug binding, providing a foundation for designing next-generation CLC-Ka inhibitors.

## Introduction

CLC (“Chloride channel”) proteins constitute a functionally heterogeneous family of plasma-membrane ion channels and intracellular transporters. By precisely regulating ion flux, they orchestrate diverse physiological processes, ranging from electrical excitability to cellular homeostasis (1, 2). In the kidney, two channel isoforms, CLC-Ka and CLC-Kb, play key roles in transepithelial salt and water homeostasis (3–6). CLC-Ka, found in the thin ascending limb of the nephron, establishes and sustains the osmotic gradient that is necessary for water reabsorption and the production of concentrated urine. Loss of CLC-Ka function dissipates this gradient, undermining the kidney’s concentrating capacity and resulting in renal diabetes insipidus (7, 8). CLC-Kb, found in the thick ascending limb and distal convoluted tubule of the nephron, provides a key pathway for chloride (Cl^−^) to leave kidney cells and return to the blood. Loss of CLC-Kb function causes Bartter syndrome type III, a severe salt wasting disorder (9). The distinct functions of CLC-Ka and CLC-Kb set them up as distinct therapeutic targets for drug development. A selective CLC-Ka inhibitor has potential to treat hyponatremia, whereas a selective CLC-Kb inhibitor has potential to act as an antihypertensive (3, 10–12). In addition to their renal roles, CLC-Ka and CLC-Kb are co-expressed in the inner ear, where their redundant functions are essential for hearing. A major challenge in developing drugs targeting CLC-Ka or CLC-Kb is the high sequence identity (91%) between the two homologs. Drug selectivity is paramount not only to target the distinct therapeutic indications of CLC-Ka and CLC-Kb, but also because simultaneous inhibition of both channels results in Bartter syndrome type IV – a condition characterized by extreme salt wasting and deafness (13–15). Here, we address the key problem of how small molecules achieve selective inhibition of CLC-Ka over CLC-Kb.

**Fig. 1.**
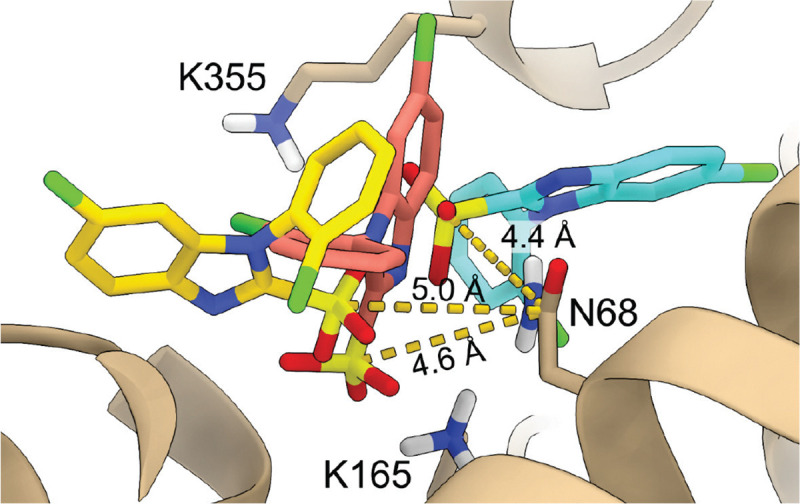
Variability in BIM1 orientation within the CLC-Ka binding pocket from prior computational docking studies. Three representative docking poses (21) are shown (in grey, salmon, and cyan) to illustrate the variability in BIM1 orientation within the pocket. Key residues N68, K165, and K355 are indicated, and distances from the closest oxygen of BIM1 to NZ atom of N68 - a major determinant of selectivity – are specified. In the present work, we introduced S68N and R355K into bovine CLC-K to reproduce the human CLC-Ka sequence at these positions, called “bCLC-Ka” this modified construct is referred to as bCLC-Ka throughout the manuscript.

Chloride-channel pharmacology has a troubled history, with classic “chloride-channel inhibitors” lacking the precision needed to distinguish between closely related channel subtypes or even unrelated ion channels (16). An oligomeric stilbene disulfonate was found to potently inhibit CLC-Ka with >100-fold discrimination against CLC-Kb, showing that selective CLC-Ka inhibition is feasible (17); however, that molecule is not drug-like and not tractable for synthetic optimization. Using insights from studies on low-potency inhibitors (12, 18), Liantonio and colleagues leveraged structure-activity relationship (SAR) analysis to develop MT-189, a low-micromolar CLC-Ka inhibitor with ~3-fold selectivity over CLC-Kb (19, 20). Building on this foundation, we designed BIM1, which retains low-micromolar potency against CLC-Ka while boosting selectivity over CLC-Kb to twenty-fold (21). Docking simulations of BIM1 into CLC-Ka homology models implicated N68 and K165 in potency, but the marked variability in the identities and orientations of BIM-interacting residues across docking solutions precluded a definitive binding-pocket map (21) (Fig. 1). An improved understanding of BIM1 binding interactions and the molecular basis of its homolog specificity is critical for designing next-generation analogs with enhanced potency and preserved CLC-Ka/CLC-Kb selectivity, suitable for evaluation in preclinical studies.

## Results and Discussion

### Experimental system

**Fig. 2.**
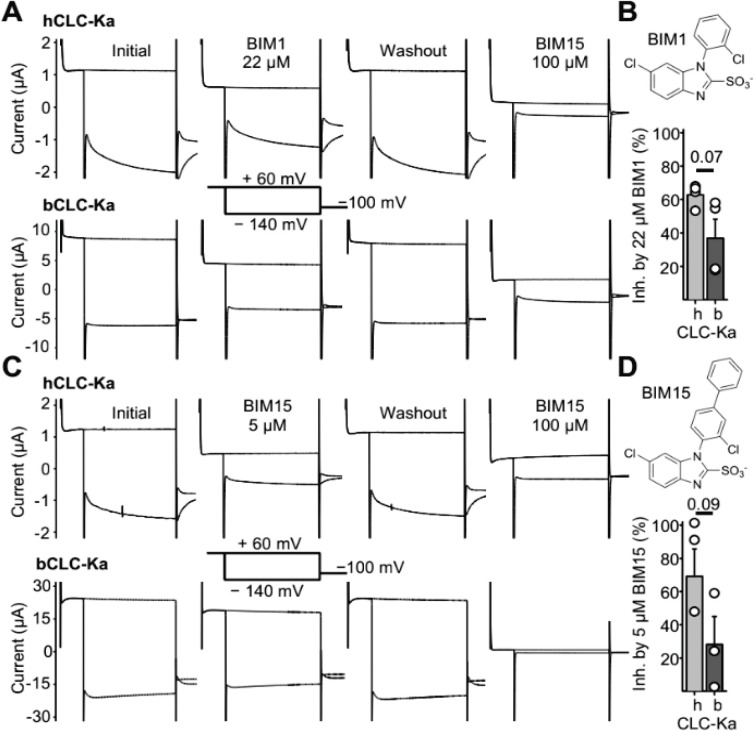
bCLC-Ka is sensitive to inhibition by BIM1 and BIM15 (*A*) TEVC recordings of oocytes overexpressing hCLC-Ka (upper traces) and bCLC-Ka (lower traces). Each set of traces show currents under four conditions: initial, after 22 μM BIM1, after BIM1 washout, and after subsequent application of 100 μM BIM15. The voltage protocol is depicted between the second set of traces. (*B*) Depiction of BIM1 chemical structure and summary data for inhibition of hCLC-Ka and bCLC-Ka by 22 μM BIM1. Bars represent the mean ± S.E.M; circles represent individual inhibition values (n = 4 biological replicates). Inhibition was calculated as described in Materials and Methods. The P-value was determined using an unpaired t-test. (*C*) TEVC recordings of oocytes overexpressing hCLC-Ka and bCLC-Ka, as in panel A except testing inhibition by 5 μM BIM15 in place of 22 μM BIM1. (*D*) Depiction of BIM15 chemical structure and summary data for inhibition of hCLC-Ka and bCLC-Ka by 5 μM BIM15. Bars represent the mean ± S.E.M; circles represent individual inhibition values (n = 3 biological replicates). Inhibition was calculated as described in Materials and Methods. The P-value was determined using an unpaired t-test.

We chose the bovine CLC-K homolog (bCLC-K) for structure-determination because its apo cryo-EM structure has been solved, providing established expression and purification protocols (22). Bovine CLC-K is a single paralog that shares 84% sequence identity with the two human CLC-K isoforms. In the computationally predicted BIM binding pocket (Fig. 1), two residues differ between bCLC-K and human CLC-Ka (hCLC-Ka) (Fig. S1), so we introduced S68N and R355K substitutions to make the bovine sequence match hCLC-Ka at those positions; we refer to this engineered channel as bCLC-Ka. Using two-electrode voltage clamp (TEVC) recordings of *Xenopus* oocytes, we established that bCLC-Ka exhibits sensitivity to BIM1 and BIM15 comparable to that of hCLC-Ka (Fig. 2, Fig. S2).

### Sulfonate position in the BIM1-bound bCLC-Ka structure

**Fig. 3.**
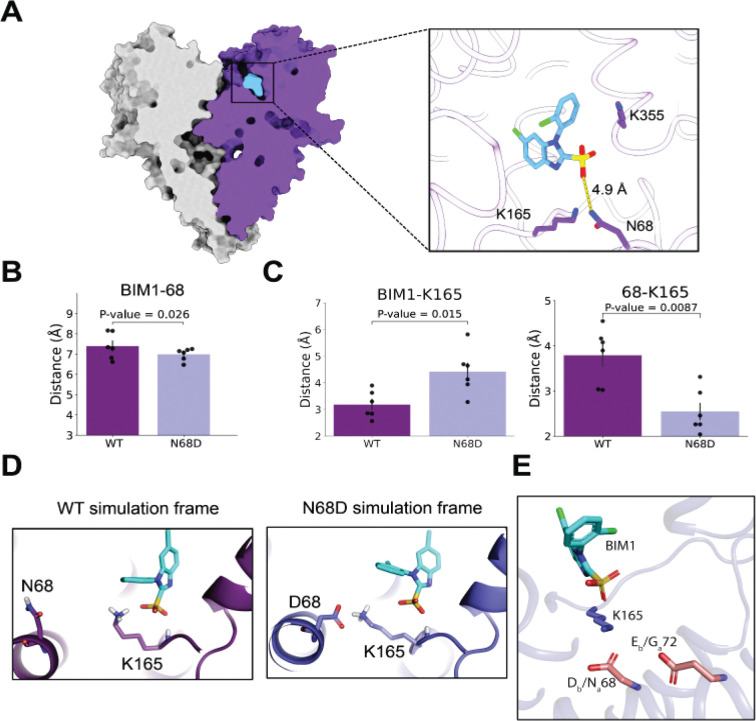
Mechanism of BIM1 CLC-K isoform selectivity. *(A)* Cryo-EM structure of bCLC-Ka bound to BIM1. The zoomed-in view of the binding pocket shows the distance between the closest oxygen of the BIM sulfonate group and the side chain nitrogen of N68. (*B*) N68 does not directly interact with the BIM sulfonate group. Bar graphs show the average distances between residue 68 (OD1 or OD2/ND2 atom) and BIM1 (closest oxygen atom) across multiple simulations. (*C*) K165 distances in the bCLC-Ka (WT) and bCLC-Ka N68D (N68D) simulations. BIM1-K165 distances are between the closest H atom on K165 and the closest oxygen on BIM1; 68-K165 distances are between the closest H atom on K165 and the closest side chain oxygen/nitrogen of N68 or D68. For panels B and C, average distances for individual simulations are shown as black dots, with black bars indicating the SEM, and P-values were assessed using the non-parametric Mann–Whitney U test. (*D*) Representative MD frames of K165 interactions. In bCLC-Ka, K165 interacts closely with the BIM1 sulfonate; in bCLC-Ka_N68D_, K165 is drawn to D68, disrupting BIM1 engagement. (*E*) E72 is positioned to contribute to BIM1 isoform selectivity. The BIM1-bound CLC-Ka structure with binding-pocket residues edited to the CLC-Kb sequence reveals pocket-facing acidic D68 and E72, replacing the neutral N68 and G72 present in CLC-Ka.

We first determined the apo structure of bCLC-Ka in nanodiscs to establish the channel architecture in a native-like membrane environment. Using an adapted bCLC-K preparation protocol (22), we obtained a 3.6 Å reconstruction (Fig. S3) that closely matches the previously reported bCLC-K structure (PDB 5TQQ), with an RMSD of 0.9 Å. We then determined the BIM1-bound structure at 2.8 Å resolution (Fig. S4). The overall architecture matches the apo structure, and BIM1 occupies the previously predicted binding site, directly occluding the chloride pathway from the extracellular side (Fig. 3A). In contrast to the apo map, which lacks well-resolved Cl^−^ densities, the BIM-bound bCLC-Ka map displays clear ion density (Fig. S5).

Prior studies of benzofuran carboxylates, as well as niflumic and flufenamic acids, established that CLC-K inhibitors typically adopt a non-coplanar arrangement of their two aromatic groups, whereas coplanar geometry is characteristic of activators (19, 20, 23). Based on predicted steric interactions between the chloro substituent and the benzimidazole ring, BIM compounds were similarly expected to adopt a non-coplanar geometry and act as inhibitors (21), and our structures confirm this. Unexpectedly, however, the sulfonate group is not positioned to form a strong interaction with N68 (Fig. 3A, right panel), a residue shown by mutagenesis to govern CLC-Ka/Kb isoform selectivity and previously predicted by molecular docking to engage directly with the ligand (21).

### MD simulations illuminate the molecular basis of BIM1 selectivity

Because prior functional data indicate that the identity of residue 68 (N68 in hCLC-Ka, D68 in hCLC-Kb) strongly influences BIM1 potency, our original hypothesis for the selectivity mechanism of BIM1 was that its sulfonate group directly interacts with N68 in hCLC-Ka but is repelled by D68 in hCLC-Kb. However, the distance between N68 and the BIM1 sulfonate in our cryo-EM structure is too far to form a strong interaction. To investigate whether the sulfonate and N68 could form transient interactions not captured in the structure, we ran molecular dynamics (MD) simulations of both wild-type bCLC-Ka and a bCLC-Ka N68D mutant (bCLC-Ka_N68D_) to represent hCLC-Ka and the Ka-to-Kb substitution respectively. Surprisingly, we found no significant difference in the distance between residue 68 and the BIM1 sulfonate between bCLC-Ka and bCLC-Ka_N68D_, and the average distance was about 7–8 Å, too far for any strong interaction to occur (Fig. 3B).

While our simulations indicate that no direct BIM1 interaction with N68 is responsible for selectivity, they suggest a different selectivity mechanism. In our simulations, the BIM1 sulfonate interacts much more closely with nearby residue K165 in bCLC-Ka than in bCLC-Ka_N68D_ (Fig. 3 C,D). This difference stems from the fact that D68 in bCLC-Ka_N68D_ frequently forms a salt bridge with K165, and formation of this bridge disrupts the ionic K165-BIM1 interaction (Fig. 3 C,D). This observation aligns with a prior CLC-Kb homology model that predicted a D68-K165 interaction (24). K165 thus likely has a more stabilizing effect on BIM1 binding to CLC-Ka than CLC-Kb. This interaction is also consistent with mutagenesis implicating K165 in BIM1 sensitivity (21).

Therefore, the selectivity mechanism for BIM1 is not driven by a direct interaction with residue 68; rather, it arises from altered BIM1 interaction with K165, which can engage BIM1 more readily in hCLC-Ka than in hCLC-Kb. This mechanism also explains why the D68N substitution in hCLC-Kb has only a modest impact on BIM1 potency compared to the reciprocal N68D change in hCLC-Ka (~3-fold vs ~13-fold (21)). hCLC-Kb contains an additional negatively charged residue, E72, one helical turn from D68, whereas hCLC-Ka has a neutral glycine at this position. Neutralizing D68 leaves E72 available to sequester K165 away from BIM1 in hCLC-Kb (Fig. 3E), providing a structural rationale for why the D68N substitution alone does not confer full BIM1 sensitivity. This model predicts that the hCLC-Kb double mutant D68N/E72G should restore BIM1 sensitivity to hCLC-Ka-like levels. Direct functional testing is precluded because neither this mutant nor the more conservative D68N/E72Q variant produced measurable currents in *Xenopus* oocytes. Even so, prior work on negatively charged inhibitors showed that mutating either D68 or E72 enhances potency in hCLC-Kb (25), implicating both residues in modulating inhibitor sensitivity and supporting the overall mechanism of BIM isoform selectivity.

### Conformational flexibility of BIM1 aligns with structure-activity potency trends

In examining the BIM1 binding pose obtained from the cryo-EM structure, we noted that several features of the published BIM SAR data (21) could not be easily reconciled. For instance, introducing bulky phenyl groups to BIM1 at two distinct positions either improved or did not alter BIM1 potency, despite these positions appearing sterically occluded in the modeled pose (Fig. S6 A,B). To investigate this discrepancy, we analyzed trajectories of our all-atom MD simulations with BIM1 bound to bCLC-Ka. These simulations revealed that the BIM1 binding pose is dynamic, rather than adopting a single discrete conformation. In particular, the ligand orientation can vary substantially (Fig. S6B). Within this ensemble, we identified specific poses that create sufficient space to accommodate bulky substituents, thereby explaining the observed SAR potency trends.

### Extracellular density and atropisomeric poses in the BIM15-bound structure

**Fig. 4.**
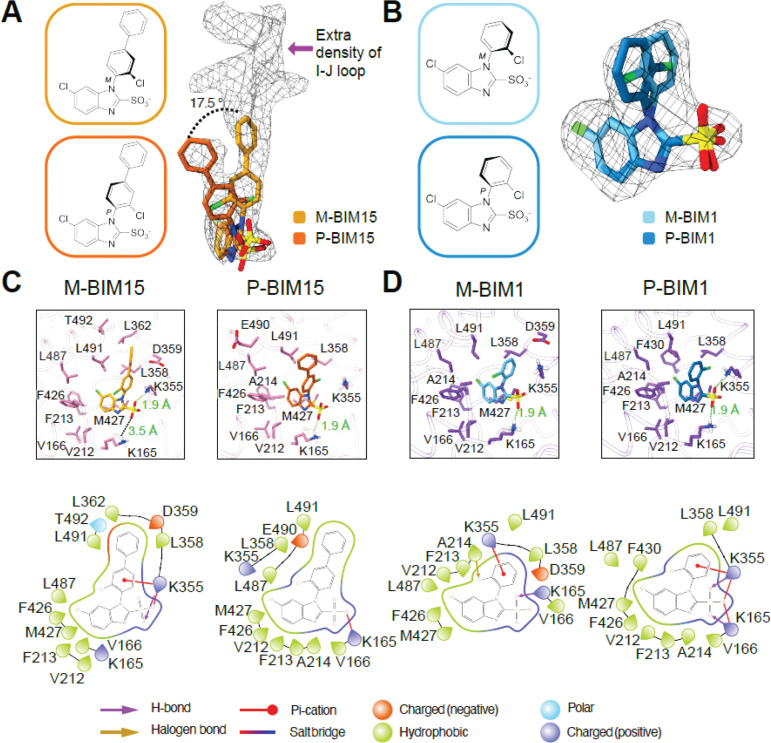
Cryo-EM reveals extra BIM15 density and resolves dual atropisomeric binding modes for BIM15 and BIM1. (*A*) Cryo-EM density for BIM15 overlaid with the molecular models of the two distinct conformations, atropisomers M-BIM15 (gold) and P-BIM15 (orange). The two atropisomers are 17.5 ° tilted relative to each other. The Q-scores (0.69 for each) indicate that both atropisomers are comparably supported by the cryo-EM density. Extra density appears above the M-BIM15 conformation. (*B*) Cryo-EM density for BIM1 overlaid with the molecular models of M-BIM1 (cyan) and P-BIM1 (blue). The Q-scores (0.72 and 0.74 for M- and P-BIM1 respectively) indicate that both atropisomers are comparably supported by the cryo-EM density. M-BIM1 corresponds to the conformation shown in Fig. 3. (*C*) *Top panels:* Views of the binding site showing Interacting residues within 4 Å of M-BIM15 and P-BIM15. *Bottom panels:* Corresponding 2D interactions plots generated using the ligand interaction diagram in Maestro (Schrödinger Release 2024.2). (*D*) *Top panels:* Views of the binding site showing Interacting residues within 4 Å of M-BIM1 and P-BIM1. *Bottom panels:* Corresponding 2D interactions plots generated using the ligand interaction diagram in Maestro (Schrödinger Release 2024.2). The raindrop shape indicates the direction the sidechain is pointing.

To further probe the basis of BIM inhibitor selectivity, we determined the structure of bCLC-Ka bound to BIM15 (Fig. S7), which is slightly more potent than BIM1 but lacks CLC-Ka/CLC-Kb selectivity (21). A prominent feature of the BIM15-bound map is the pronounced region of extra density above the ligand toward the extracellular side (Fig. 4A). We propose that this density originates from residues in the extracellular I-J loop of bCLC-Ka, which is not sufficiently resolved to model. Focusing on the ligand, we find that BIM15 occupies the binding pocket in two distinct conformations. One conformation matches the pose observed for BIM1. The second maintains the non-coplanar geometry but is rotated such that the chloro substituent projects in the opposite direction (Fig. 4A). Following the nomenclature of Bringmann et al. (26), these two poses correspond to the M- and P-atropisomers of BIM15. Because the two BIM15 poses were so unambiguously resolved, we reexamined the BIM1 density and found that it is best fit by two atropisomeric conformations (Fig. 4B), although the difference between its poses is more subtle than for BIM15, whose atropisomers differ by a 17.5° tilt.

### Conserved BIM interactions and the structural basis of BIM15’s lack of isoform selectivity

Structural analysis of the binding site (Fig. 4C,D) shows that both BIM1 and BIM15 inhibitors, including their respective atropisomers, are anchored to bCLC-Ka through a conserved polar network and a shared hydrophobic pocket. The sulfonate forms critical hydrogen-bonding interactions with K165 and K355, while the benzimidazole core is stabilized by hydrophobic contacts with V166, V212, F213, A214, F426, M427, and L487, and the chlorobenzene ring interacts with L358 and L491. Although the overall binding modes are conserved between the atropisomers, they show distinct residue-level interactions. For BIM1, the primary distinction is that the M-isomer’s chlorobenzene is slightly tilted towards D359 in the upper sub-pocket. Compared to BIM1, BIM15 occupies a larger structural footprint, and its atropisomers differ more markedly, with M-BIM15 adopting an upward orientation that enables unique contacts with D359, L362, and T492. Using PDBePISA analysis to quantify the ligand-protein interaction area (see Methods), the BIM1 atropisomers show nearly identical interaction surface areas (325.9 Å^2^ for M-BIM1 and 332.4 Å^2^ for P-BIM1), whereas BIM15 exhibits a larger atropisomeric difference, with interface surface areas of 391.3 Å^2^ for M-BIM15 and 370 Å^2^ for P-BIM15. Notably, the M-BIM15 interface area is likely underestimated, as the unresolved I-J loop leaves potential loop contacts out of the calculation.

Analysis of the M-BIM15 conformation establishes the molecular basis for BIM15’s lack of isoform selectivity between CLC-Ka and CLC-Kb (13). M-BIM15 shows a weakened sulfonate-K165 interaction, with an O-H distance of 3.5 Å compared to 1.9 Å in the other BIMs; instead, the sulfonate is stabilized by K355 (1.9 Å O-H distance) (Fig. 4 C,D). As a result, the D68 induced displacement of K165 in CLC-Kb has no functional consequence: the absence of a K165 interaction, combined with stabilization by K355, effectively nullifies the K165-dependent selectivity mechanism used by BIM1 and allows BIM15 to inhibit CLC-Kb as efficiently as CLC-Ka. This change in interaction may be driven by BIM15’s engagement with the I-J loop, which sits directly above M-BIM15 (Fig. 4A) and appears to pull the inhibitor out of the K165-stabilized position

### Hydrophobicity differences between benzofurans and BIM inhibitors

In developing BIM inhibitors from the less selective but similarly shaped benzofuran series, homology-model docking suggested that CLC-Kb presents a more hydrophobic binding site than CLC-Ka (21). This observation led us to propose that the lower hydrophobicity of the BIM scaffold contributes to its improved selectivity over the benzofurans in the absence of additional stabilizing mechanisms such as engagement of the I-J loop in BIM15 binding. Electrostatic calculations based on the structures determined here support this idea, revealing a similar Ka/Kb difference in the hydrophobicity of the BIM1 binding pocket (Fig. S8). This hydrophobicity-driven contribution to selectivity complements the mechanism outlined above and provides additional context for understanding isoform selectivity of CLC-K channel inhibitors.

### The conformationally flexible I-J loop interacts with bound BIM1 and BIM15

**Figure 5:**
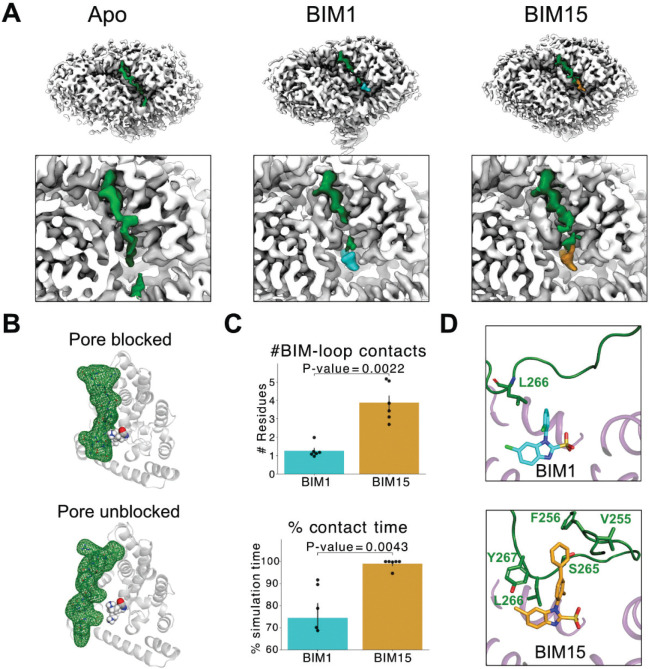
The conformationally flexible I-J loop blocks the pore and interacts with bound BIM1 and BIM15. (*A*) Top views of cryo-EM density for apo, BIM1-bound, and BIM15-bound bCLC-Ka. Upper panels show the full protein; lower panels provide zoomed-in views of the extracellular region. All maps are at the same threshold: 7 standard deviations from the mean. BIM1 and BIM15 densities are shown in cyan and gold respectively. The I–J loop (dark green) spans ~40 Å between helices and is the only extracellular segment unresolved in our models, supporting its assignment across all three structures. (*B*) Two representative simulation frames from the apo bCLC-Ka simulation, one where the I-J loop blocks the pore, and one where it does not. The I-J loop is displayed as dark green mesh with sticks inside showing the residues. BIM-interacting residues 165, 166, and 355 are displayed as spheres with CPK coloring, marking the position of the channel pore. (*C*) Quantification of contacts to the I-J loop (residues 255–277). *Top:* Average number of loop residues contacting BIM1 or BIM15 (4 Å cutoff) *Bottom:* Contact-time percentage, calculated as the fraction of frames with any loop–ligand contact (4 Å cutoff). For both plots, average values for each individual simulation are shown as black dots, and black bars represent the SEM. P-values were calculated using the non-parametric Mann-Whitney U test. (*D*) MD snapshots showing representative interactions of the I-J loop with BIM1 (top panel) and BIM15 (bottom panel). All residues within 4Å of BIM1/BIM15 are shown. In the representative simulation frames, only one I-J loop residue is in contact with BIM1, while five are in contact with BIM15.

Across all our bCLC-Ka structures – apo, BIM1-bound, and BIM15–bound – we detect density corresponding to the extracellular I-J loop (Fig. 5A), though in each case the resolution is insufficient for confident modeling. This region is noteworthy because it appears poised to influence channel gating. In our structures, as well as in the previously reported bCLC-K structure (22), the loop drapes over the pore entryway, positioning it as a plausible gating element. Consistent with this idea, mutagenesis studies have suggested that residues E261 and E278 within the I-J loop form a Ca^2+^-binding site essential for Ca^2+^-dependent gating (27, 28).

Seeing extra density in the BIM15–bound structure and identifying the unresolved I-J loop as the most plausible source (Figs. 4A, 5A), we examined MD simulations of bCLC-Ka in which the loop was modeled to the extent permitted by the density. With BIM1 bound, the simulations showed the I-J loop as highly flexible, sampling positions that intermittently blocked access to the BIM1 site (Fig. S9). In apo simulations, the loop was similarly dynamic and moved down into the BIM1 binding pocket, where it intermittently blocked the channel pore (Fig. 5B). The loop’s flexibility and its incursions into the BIM site prompted us to ask whether, and how, it interacts directly with bound BIM molecules. To investigate this, we quantified I-J loop contacts with both BIM1 and BIM15. With BIM1, loop residues made only transient and heterogeneous contacts, with no single dominant interaction pattern across the different simulations. In contrast, BIM15 exhibited a recurring interaction with residue L266 in 96% ± 6% of frames and, on average, contacted more loop residues than BIM1 (Fig. 5C). In other words, BIM15 interacted with more residues on the I-J loop simultaneously versus BIM1. These increased contacts likely explain why I-J loop density is continuous with the BIM density in the BIM15-bound cryo-EM map but not in the BIM1-bound structure, and they may contribute to BIM15’s slightly higher potency (55 ± 7% vs. 38 ± 3% inhibition at 5 μM (21)). This conclusion that I-J loop engagement contributes to potency aligns with studies showing that I-J loop mutations strongly influence the modulation of CLC-Ka channel activity by niflumic acid, a nonselective chloride-channel inhibitor (29). Representative snapshots of the loop-ligand interactions for BIM1 and BIM15 are shown in Fig. 5D.

### The I-J loop mediates Ca^2+^-dependent gating

Extracellular Ca^2+^ strongly activates CLC-K channels in both native and heterologous systems (27, 30). Currents increase ~3-fold when Ca^2+^ rises from 100 μM to 2 mM and another ~4-fold at 50 mM Ca^2+^ (27). Because CLC-K channels are maximally responsive near physiological concentrations (~2 mM), and extracellular [Ca^2+^] varies across nephron segments, this modulation is likely to be functionally significant in vivo (27, 31, 32). To understand the molecular basis of this effect, an extensive mutagenesis campaign identified residues E261 and E278 on the I-J loop as essential for this Ca^2+^-dependent activation, and noise analysis showed that Ca^2+^ enhances current by influencing channel gating rather than single-channel conductance (27). Together, these findings implicate the I-J loop in Ca^2+^-dependent gating and motivate a structural analysis of how Ca^2+^ binding influences this region.

**Fig. 6.**
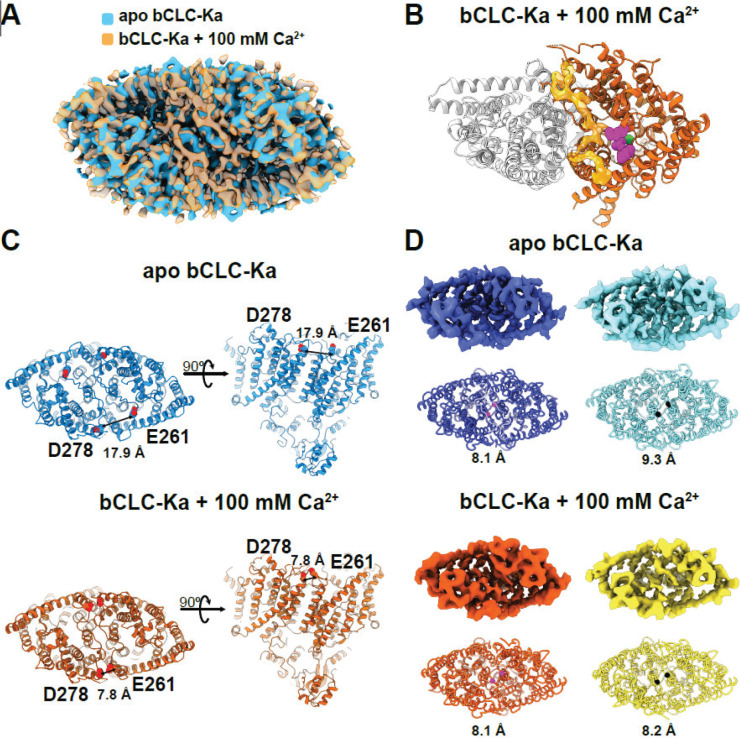
Calcium orders the I-J loop away from the pore. (*A*) Overlay of cryo-EM maps of apo bCLC-Ka (no added Ca^2+^; blue) and bCLC-Ka with 100 mM Ca^2+^ (gold) reveals additional I–J loop density in the Ca^2+^-bound map. Both maps are sharpened and shown at a threshold of 8 standard deviation above means. (*B*) Cryo-EM structure of bCLC-Ka in the presence of 100 mM Ca^2+^. The high-quality density for the I-J loop (orange) supports unambiguous model building of this segment. BIM-interacting residues 165, 166, and 355 are displayed as pink spaced-filled sidechains on the orange subunit, marking the position of the channel pore. (*C*) Ca^2+^ draws E261 and E278 together. In the apo structure (blue), the Cα-Cα distance between the residues is >17 Å, whereas in the 100 mM Ca^2+^ structure (orange), the corresponding Cα-Cα distance contracts to 7.8 Å. (*D*) GMM analysis shows asymmetric subunit motion in apo bCLC-Ka and synchronized, concerted motion in bCLC-Ka with 100 mM Ca^2+^. The top panels show the first (left) and last (right) density maps along the principal motion pathway identified in GMM latent space. The bottom panels show the corresponding structural models, with residue Q494 marked as pink (first snapshot) and black (last snapshot) spheres. In apo bCLC-Ka, the distance between residues changes over the trajectory, whereas in Ca^2+^-bound bCLC-Ka it remains constant, reflecting asymmetric versus synchronous subunit motion.

Given the pronounced flexibility of the I–J loop and its ability to drape over the pore and inhibitor-binding site, we hypothesized that Ca^2+^ binding stabilizes the I-J loop in a single conformation positioned away from the pore and the BIM binding site. To test this, we solved the cryo-EM structure of bCLC-Ka in the presence of 100 mM Ca^2+^ (Fig. S10). The resulting map, at 3.4 Å resolution, shows markedly improved density for the I-J loop (Fig. 6A), supported by a higher Q-score (Fig. S10E) compared to the 0 mM Ca^2^ structure (Fig. S3E). The ordered loop density runs along the subunit interface and is clearly displaced from the pore (Fig. 6B). Although Ca^2+^ is not directly visualized, its presence draws the proposed chelating residues, E261 and E278 into proximity, reducing their Cα–Cα distance from 17.9 Å to 7.8 Å (Fig. 6C). Nearby residues E259 and E281, previously implicated in tuning CLC-Ka’s calcium sensitivity (28), do not appear positioned to directly coordinate Ca^2+^ with E261 and E278, but may contribute through a general electrostatic effect. Together, these structural changes provide direct evidence that Ca^2+^ gates the channel by stabilizing the I-J loop in a conformation that no longer occludes the pore.

### Ca^2+^ enhances coordinated motion in CLC-K channels

To determine how Ca^2+^ binding shapes conformational dynamics, we applied Gaussian Mixture Model (GMM) variability analysis (33) to extract the structural flexibility at the transmembrane domain from our cryo-EM data. While conformational changes were detected in both conditions, the analysis revealed pronounced differences in subunit coupling in bCLC-Ka particles treated with or without Ca^2+^. In the absence of Ca^2+^, the two subunits follow distinct trajectories and move in different directions, consistent with partially independent behavior. Under high Ca^2+^, however, the subunits become dynamically locked and move together in a coordinated fashion (Fig. 6D, Fig. S11, supplementary movies). This Ca^2+^-dependent transition from decoupled to concerted motion suggests that Ca^2+^ binding reorganizes the energy landscape and may favor a cooperative gating process, akin to the common gating described in CLC-0 (34, 35) and inferred but not fully defined in other homologs, including CLC-K channels (1, 36). Thus, GMM analysis reveals that Ca^2+^ binding modulates intersubunit coupling, motivating future work to determine whether these Ca^2+^-dependent changes contribute to the poorly understood common gating of CLC-K channels.

### Comparison with previously reported class-2 structure

None of the structures determined in this study match the alternative “class-2” structure reported in the original bCLC-K cryo-EM study (PDB 5TR1). That class-2 structure resembles both the main class-1 structure from the original study (PDB 5TQQ) and the conformations resolved here, but it features a slightly looser dimer interface and a ~6° tilt between the two transmembrane domains (22). Given similar particle numbers between studies, which reduces the likelihood that sampling depth explains the discrepancy, we hypothesize that the domain-tilted conformation may reflect the presence of detergent and a Fab fragment in the earlier dataset, neither of which was present in our preparations. Thus, the conformations resolved here represent the structural states accessible under our experimental conditions, with a dimer interface similar to that seen in other CLC channels, and they place CLC-Ka’s ligand interactions within a well-defined structural framework.

## Conclusions

CLC-Ka is an attractive therapeutic target for hyponatremia, a common water-balance disorder that remains difficult to manage and often impairs quality of life (37–39). Our structural and computational analyses reveal how benzimidazole inhibitors engage the CLC-Ka binding pocket, showing that interactions with a conserved lysine are central to both inhibition and isoform selectivity, while surrounding residues modulate these contacts. We also identify a dynamic extracellular loop whose movements shape access to the binding pocket and couple to Ca^2+^-dependent gating. Together, these findings unify ligand binding with channel gating and explain how subtle sequence differences translate into distinct pharmacological outcomes, providing a mechanistic framework for designing next-generation CLC-Ka inhibitors and advancing therapeutic strategies targeting renal chloride channels.

## Materials and Methods

### Constructs for CLC expression in Xenopus oocytes

Oocyte expression constructs for bovine CLC-K and Barttin were obtained from the MacKinnon laboratory (22). To mimic the human CLC-Ka BIM-binding site, two mutations, S68N and R355K, were introduced into the bovine CLC-K construct. Constructs for human CLC-Ka and Barttin were used as previously described (21). cRNA was generated using linearized DNA templates and the T7 mMessage mMachine Kit (Life Technologies)

### Chemical synthesis

BIM1 and BIM15 were prepared as described in Koster et al. (21)

### Electrophysiological measurements of oocytes expressing CLC-K channels

Electrophysiology experiments for Figure 2 were performed on a fee-for-service basis by NMI Technologie Transfer GmbH. Additional in-house recordings on CLC-Ka reproduced the results shown in Figure 2. Attempts to express and record from the two CLC-Ka mutants (D68N/E72G and D68N/E72Q) were also performed in-house, but currents were not distinguishable from background. Overall methods and instrumentation were the same for both, except that in-house experiments were performed with manual rather than automated oocyte injection

Oocytes from *Xenopus laevis* (Ecocyte Bio Science) were separated and transferred to either a 35-mm culture dish (in-house measurements) or to a 96-well plate with a conically shaped well bottoms (NUNC), prefilled with Barth’s medium containing 88 mM NaCl, 1 mM KCl, 0.4 mM CaCl_2_, 2.33 mM Ca(NO_3_)_2_, 2.4 mM MgSO_4_, 2.4 mM NaHCO_3_, 5 mM Tris-HCl at pH = 7.6 adjusted with NaOH. Oocytes, were subsequently injected with 5, 10, or 20 ng of hCLC-Ka/Barttin (2:1) RNA mix, or 3.6 ng of RNA of the bCLC-Ka/Barttin (2:1) mix, using either manual injection (Drummond Nanoject II) or using a RobooInject System (Multichannel Systems, Reutlingen, Germany) (NMI measurements) and stored for 1 day (bCLC-Ka) or 2 – 4 days (hCLC-Ka) after injection.

Automated two-electrode voltage clamp (TEVC) recording was performed on a Roboocyte2 setup (Multichannel Systems, Reutlingen, Germany). Before the measurements, the oocytes were perfused with ND96 medium (96 mM NaCl, 2 mM KCl, 1 mM MgCl_2_, 1.8 mM CaCl_2_ and 5 mM Na-Hepes at pH = 7.6 adjusted with NaOH). The microelectrodes within the measuring heads (Multichannel Systems) were filled with 3 M KCl. Upon oocyte impalement, the holding potential was adjusted to the transmembrane potential ($\Phi_{M}$). Oocytes with $\Phi_{M}\;>\;–\;10\;mV$ (less negative) were excluded from the measurements, since these values indicate leaky oocytes.

Ion channel currents were assessed in one-minute intervals by a pair of two sequential voltage-clamp recordings that share the same prepulse and tail pulse but differed in their test pulse. Each protocol consistent of a 60-mV prepulse for 50 ms, followed by either a − 140-mV test pulse (recording 1) or 60-mV test pulse (recording 2) for 200 ms, and a final tail pulse of − 100 mV for 50 ms. For CLC-Ka, currents in response to the − 140-mV test pulse served as a quality control because this voltage produced the channel’s characteristic increase in current over the 200-ms test pulse. To quantify this gating behavior, the parameter g was calculated as:

(1) $$g=\frac{I_{ss}-I_{0}}{I_{ss}}$$

Where $I_{0}$ is the current in the beginning of the −140-mV pulse and $I_{ss}$ the current in the end of the pulse. Recordings with gating parameters below 0.3 were considered to represent non-CLC-Ka currents and were not advanced to inhibitor testing. For bCLC-Ka, which lacks voltage-dependent gating, this metric could not be applied. Instead, oocytes with currents < 800 nA or > 30 μA at 60 mV were not used, as such values indicate either low bCLC-Ka expression or leaky oocytes.

### Calculation of the CLC-K inhibition by BIM1 and BIM15

Inhibition values for BIM1 and BIM15 were determined using a four-step protocol: (1) Initial current (**I**_**init**_), recorded before inhibitor application. (2) Current recorded after the perfusion of 22 μM BIM1 or 5 μM BIM15 (**I**_**c**_). (3) Current after the washout of the inhibitors (**I**_**WO**_) by continuous perfusion of ND96 until I_WO_ returned to 80 – 120 % of I_Init_; experiments that did not meet this washout criterion were not advanced. (4) Current recorded after the perfusion of 100 μM BIM15 (**I**_**L**_), which fully inhibits hCLC-Ka currents (Koster et al. 2018). Perfusion was performed at 1 mL/min for 3 min (steps 1, 2 and 4) or > 3 min (step 3). Experiments proceeded only when currents stabilized such that 3 consecutive recordings remained within 5 % of the previous recording. Recordings with I_L_ > 1000 nA were excluded, since this level of leak indicates currents with insufficient specificity for CLC-K channels.

The inhibition was calculated according to:

(2) $$Inhibition(\%)=100\cdot \left(1-\frac{I_{C}-I_{L}}{I_{0}-I_{L}}\right)$$

$I_{0}$ is the uninhibited current measured either before inhibitor application ($I_{init}$) or after washout ($I_{wo}$). For each experiment, 2 inhibition values were therefore calculated – one using $I_{init}$ and one using $I_{WO}$. The final inhibition value for that experiment was obtained by averaging these two values. Results are displayed as the mean ± SD of at least three independent measurements and the statistical significance calculated using an unpaired t-test.

### Protein expression and purification

The bCLC-K construct used for protein production for structural studies was obtained from the MacKinnon laboratory (22). To mimic the human CLC-Ka BIM-binding site, mutations S68N and R355K were introduced into the construct, generating “bCLC-Ka.”. The bCLC-Ka construct was transformed into DH10Bac competent cells (Invitrogen) to generate recombinant bacmid DNA. Purified bacmid was transfected into Sf9 cells using Cellfectin-II (Invitrogen) to produce baculovirus. The baculovirus was further amplified twice in Sf9 cells. The protein was expressed in HEK293S GnTI^−^ cells with the amplified baculovirus. HEK293S GnTI^−^ (ATCC: CRL-3022) cells were cultured in Freestyle 293 medium (Invitrogen) supplemented with 2% fetal bovine serum on a shaker at 37°C in the presence of 8% CO_2_ to a density between 2– 3 × 10^6^ cells per mL, then infected with 2% vol/vol baculovirus. After culturing for another 8–16 hours, sodium butyrate was added at final concentration of 10 mM, then further expressed for 48 hours at 37°C before harvest. Cells were harvested by centrifugation and stored at −80°C until use.

2 L of frozen cell pellets were resuspended in resuspension buffer containing 50 mM 4-(2-hydroxyethyl)-1-piperazineethanesulfonic acid (HEPES), pH 7.5, 300 mM NaCl, and protease inhibitor cocktail tablet (MedChem Express), and lysed with 20 strokes of a Dounce homogenizer. Cellular debris were collected by centrifugation at 18,000 rpm for 40 min at 4°C, and then resuspended with resuspension buffer supplemented with 1% n-dodecyl-ß-D-maltoside (DDM) and 0.1% cholesteryl semisuccinate (CHS). After extraction for 2 hours, the lysate was centrifuged at 18,000 rpm for 40 min at 4°C. The clarified lysate was incubated with cobalt resin (TAKARA) for 1 hour at 4°C. Resin was washed with wash buffer containing 50 mM HEPES, pH 7.5, 300 mM NaCl, 10 mM Imidazole, 0.04% DDM, and 0.004% CHS. Purified protein was released from resin with 100 μg HRV 3C protease and incubated at 4°C for overnight. The retrieved protein was concentrated to 0.5 mL with Amicon Ultra (50 kDa cutoff, EMD Millipore) and followed by size-exclusion chromatography (SEC) using a superdex 200 Increased 10/300 chromatography column on an AKTA FPLC system (Cytiva) with buffer containing 20 mM HEPES, 100 mM NaCl, 1 mM DTT, 0.5 mM EDTA, 0.04% DDM, and 0.004% CHS. Protein fractions were pooled, concentrated with Amicon Ultra (50 kDa cutoff, EMD Millipore) to ~1.5–2.5 mg/mL.

### Cryo-EM sample and grid preparation

All bCLC-Ka cryo-EM structures were determined in Salipro lipid nanoparticles (40) following previously established protocols (41). Purified bCLC-Ka in DDM (1.5–2.5 mg/mL) was mixed with 10 mM POPC, 10 mM POPG, and 1.5 mM Saposin A in incorporation-buffer (50 mM Tris-HCl, pH 7.6, and 150 mM NaCl) to a final volume of 4 mL. The final reaction mixture contained 0.3 mg/mL bCLC-Ka, 1.35 mM POPC, 450 μM POPG, and 120 μM Saposin A. After incubation at 4 °C for 40 min, 50% (w/v) Amberlite XAD-2 (MilliporeSigma) was added for 15 min at 4 °C to remove the detergent. The beads were removed by centrifugation, and the mixture was concentrated to approximately 250 μL using a 10 kDa molecular weight cutoff (MWCO) Amicon centrifugal filter (MilliporeSigma). The sample was further purified by size-exclusion chromatography (SEC) using a Superdex 200 Increase 10/300 GL column (Cytiva) equilibrated in incorporation buffer.

Fractions containing bCLC-Ka in Salipro (eluting at ~12.3 mL) were pooled and concentrated using a 30 kDa MWCO Amicon filter to a final A280 of 2–4. For ligand-bound states, 1 mM BIM1/BIM15 or 100 mM CaCl_2_ was added and incubated on ice for 2 hours. Prior to grid preparation, 1 mM fluorinated FOS-Choline-8 was added to improve ice thickness and mitigate preferred orientation. For vitrification, 3 μL of the sample was applied to glow-discharged Quantifoil R1.2/1.3 Cu 200-mesh grids, blotted for 3 s with Whatman No. 1 filter paper, and plunge-frozen in liquid ethane using a Vitrobot Mark IV (Thermo Fisher Scientific) at 4 °C and 100% humidity.

### Cryo-EM data acquisition

All cryo-EM data were collected on a Thermo Fisher Scientific Titan Krios cryo-electron microscope operating at 300 kV. The apo bCLC-Ka dataset was acquired using a Falcon 4 direct electron detector without an energy filter. The other three datasets (BIM1, BIM15, and 100 mM Ca^2+^) were collected using a Falcon 4 detector and a Selectris energy filter with a slit width of 10 eV. Data collection parameters are summarized in Table 1. Automated data collection was performed using the EPU software (Thermo Fisher Scientific).

### Cryo-EM image processing

The complete data processing workflows, including specific details for each sample and dataset, are reported in Figures S3, S4, S7, and S10. For all four datasets (apo, BIM1, BIM15, and 100 mM Ca^2+^), the raw movies were pre-processed (motion correction and CTF estimation) in CryoSPARC Live. Micrographs were curated based on defined criteria, including total motion, CTF fit resolution, and relative ice thickness. Initial particle picking was performed using a blob picker. The resulting particles were pruned through multiple rounds of 2D classification, ab initio reconstruction, and heterogeneous refinement in CryoSPARC. Particles that yielded maps with recognizable protein features were then used for template picking (for the apo dataset) or to train a Topaz particle picker (for the BIM15 and 100 mM Ca^2+^ datasets). Several rounds of heterogeneous refinement (using three classes) were applied to clean the re-picked particle stacks. For the apo dataset, 3D classification without alignment was also performed in RELION, using a mask covering only the protein density for further cleanup. The final reconstructions were generated using non-uniform refinement in CryoSPARC with C2 symmetry imposed.

For the GMM based structure heterogeneity analysis, final particles and their assigned poses from the two Cryo-EM datasets (bCLC-Ka with 0 or 100 mM added Ca^2+^) were imported into EMAN2. Iterative GMM based orientation refinements using a mask focusing on the transmembrane domain (33) were performed to exclude the heterogeneity of the CTD domains and improve the local resolution of the transmembrane domains. The deep learning-based heterogeneity analysis was then applied to the particles with a mask focusing on the transmembrane domains and using information up to 5 Å resolution. The analysis revealed the conformational changes of the region, and the first two eigen trajectories from the analysis were shown for each dataset.

### Model building and structure analysis

A bCLC-K crystal structure (PDB: 5TQQ) was used as an initial atomic model. The initial models were first modified based on our construct (i.e. serine to glutamine and arginine to lysine). These models were rigid-body docked into the cryo-EM map and refined using ISOLDE (42) and PHENIX (43). All atomic models were validated in the PHENIX validation job. Per-residue Q-score (44) was used to assess resolvability of the maps. All structure figures were prepared with UCSF ChimeraX (45). 2D interaction plots were generated using the Ligand Interaction Diagram tool in Maestro (Schrödinger Release 2024.2) with a default 4 Å distance cutoff. To prepare the structures, a protein preparation step (including bond order assignment and protonation state assignment at neutral pH) was performed using the Protein Preparation Wizard in Maestro. The ligand-protein interaction areas were calculated with PDBePISA (https://www.ebi.ac.uk/pdbe/pisa/).

Electrostatic surface maps were generated using the Adaptive Poisson-Boltzmann Solver plugin for PyMOL (Schrödinger) and are depicted as surface potentials in Fig. S8. For bCLC-Ka, we calculated potentials directly from the cryoEM structure. For CLC-Kb, we created a “bCLC-Kb” model in PyMOL by mutating all transmembrane domain residues on bCLC-Ka which were identical between bCLC-Ka and hCLC-Ka but different in hCLC-Kb to their hCLC-Kb counterparts. We then calculated potentials on the resulting structure model.

### System setup for molecular dynamics simulations

We performed simulations of bCLC-Ka with five conditions: (1) wild-type apo protein monomer, (2) wild-type monomer with BIM1 bound, (3) wild-type dimer with BIM1 bound, (4) N68D mutant monomer with BIM1 bound, and (5) wild-type BIM15 bound monomer with the I-J loop modeled in. Simulations for (1) through (4) were initiated from a structure of bCLC-Ka bound to BIM1, based on the cryo-EM data reported in this manuscript (specifically, from a model very similar to that presented here but based on an earlier refinement). The I-J loop was modeled into the BIM1-bound structure using the Model Loops function in ChimeraX, which employs the Modeller web server (46). Likewise, simulations for (5) were initiated from an earlier refinement very similar to the structure reported here of bCLC-Ka bound to BIM15. For (1), (2), (4) and (5), one protomer was deleted from the structure using Prime (Schrödinger). For (1), BIM1 was deleted from the pocket using Prime. For (4), N68 was manually changed to an aspartate in Prime. For (5), we aligned the BIM15-bound structure to the BIM1-bound structure, copied over all coordinates for loop residues 256–273, added bonds between the loop and BIM15 residues surrounding the loop, and relaxed the resulting construct using Prime. For each simulation condition, we performed six independent simulations, each between 0.85 and 1 μs in length. For each simulation, initial atom velocities were assigned randomly and independently.

For all simulation conditions, the protein structure was aligned to the Orientations of Proteins in Membranes entry for 5TQQ (bovine CLC-K) using PyMOL. Prime was used to add capping groups to protein chain termini. Protonation states of all titratable residues were assigned at pH 7. Histidine residues were modelled as neutral, with a hydrogen atom bound to either the delta or epsilon nitrogen depending on which tautomeric state optimized the local hydrogen-bonding network. Using Dabble (47), the prepared protein structures were inserted into a pre-equilibrated palmitoyl-oleoyl-phosphatidylcholine (POPC) bilayer, the system was solvated, and sodium and chloride ions were added to neutralize the system at a concentration of 150 mM. The final systems comprised approximately 70,000 atoms, and system dimensions were approximately 80×90×100 Å. Simulations were conducted in the absence of an applied transmembrane voltage.

### Molecular dynamics simulation and analysis protocols

We used the CHARMM36m force field for proteins, the CHARMM36 force field for lipids and ions, and the TIP3P model for waters. Parameters for BIM1 and BIM15 were generated using the CHARMM General Force Field (CGenFF) via the ParamChem web server (48). All simulations were performed using the Compute Unified Device Architecture (CUDA) version of particle-mesh Ewald molecular dynamics (PMEMD) in AMBER20 on graphics processing units (GPUs).

Systems were first minimized using three rounds of minimization, each consisting of 500 cycles of steepest descent followed by 500 cycles of conjugate gradient optimization. 10.0 and 5.0 kcal∙mol^−1^∙Å^−2^ harmonic restraints were applied to the protein and lipids for the first and second rounds of minimization, respectively. 1 kcal∙mol^−1^∙Å^−2^ harmonic restraints were applied to the protein for the third round of minimization. Systems were then heated from 0 K to 100 K in the NVT ensemble over 12.5 ps and then from 100 K to 310 K in the NPT ensemble over 125 ps, using 10.0 kcal∙mol^−1^∙Å^−2^ harmonic restraints applied to protein heavy atoms. Subsequently, systems were equilibrated at 310 K and 1 bar in the NPT ensemble, with harmonic restraints on the protein non-hydrogen atoms tapered off by 1.0 kcal∙mol^−1^∙Å^−2^ starting at 5.0 kcal∙mol^−1^∙Å^−2^ in a stepwise fashion every 2 ns for 10 ns, and then by 0.1 kcal∙mol^−1^∙Å^−2^ every 2 ns for 20 ns. Production simulations were performed without restraints at 310 K and 1 bar in the NPT ensemble using the Langevin thermostat and the Monte Carlo barostat and using a timestep of 4.0 fs with hydrogen mass repartitioning. Bond lengths were constrained using the SHAKE algorithm. Non-bonded interactions were cut off at 9.0 Å, and long-range electrostatic interactions were calculated using the particle-mesh Ewald (PME) method with an Ewald coefficient of approximately 0.31 Å^−1^, and 4th order B-splines. The PME grid size was chosen such that the width of a grid cell was approximately 1 Å. Trajectory frames were saved every 200 ps during the production simulations. The AmberTools17 CPPTRAJ package was used to reimage trajectories (49). Simulations were visualized and analyzed using Visual Molecular Dynamics (VMD) (50) and PyMOL.

## Supplementary Material

Supplement 1

## Figures and Tables

**Table 1.** Cryo-EM data collection, refinement and validation statistics

	bCLC-Ka	bCLC-Ka BIM1		bCLC-Ka BIM15		bCLC-Ka 100 mM Ca^2+^

**Data collection and processing**						
Magnification	96,000	165,000		165,000		165,000
Voltage (kV)	300	300		300		300
Electron exposure (e−/Å^2^)	60	60		60		60
Defocus range (μm)	−0.8 to −2	−0.8 to −2		−0.8 to −2		−0.8 to −2
Pixel size (Å)	0.82	0.74		0.74		0.74
Symmetry imposed	C2	C2		C2		C2
Initial particle images (no.)	3,543,443	3,229,270		421,850		328,926
Final particle images (no.)	74,770	248,372		271,313		191,200
Map resolution (Å)	3.6	2.8		3.0		3.4
FSC threshold	0.143	0.143		0.143		0.143
Map resolution range (Å)	2.1 to 11.1	1.6 to 8.8		1.8 to 8.4		2.2 to 11.3
**Refinement**		P-BIM1	M-BIM1	P-BIM15	M-BIM15	
Initial model used (PDB code)	5TQQ	5TQQ	5TQQ	5TQQ	5TQQ	5TQQ
Model resolution (Å)	3.5	2.7	2.7	2.9	2.9	3.3
FSC threshold	0.143	0.143	0.143	0.143	0.143	0.143
Map sharpening *B* factor (Å ^2^)	−116.8	−110.3	−119.1	−119.5		
Model composition						
Non-hydrogen atoms	9590	9356	9356	9346	9346	9592
Protein residues	1242	1208	1208	1206	1206	1242
Ligands		6	6	6	6	2
*B* factors (Å ^2^)						
Protein	46.31	43.08	44.22	53.27	38.62	72.17
Ligand		63.38	64.21	85.94	62.64	
R.m.s. deviations						
Bond lengths (Å)	0.002	0.014	0.014	0.013	0.013	0.021
Bond angles (°)	0.494	1.892	1.867	1.880	1.773	1.663
**Validation**						
MolProbity score	1.30	1.24	1.17	0.57	1.08	1.34
Clashscore	4.56	2.29	1.92	0.00	1.86	3.57
Poor rotamers (%)	1.07	0.80	0.40	0.50	0.20	1.17
Ramachandran plot						
Favored (%)	97.80	96.49	96.66	97.65	97.32	97.24
Allowed (%)	2.20	3.51	3.34	2.35	2.68	2.76
Disallowed (%)	0.00	0.00	0.00	0.00	0.00	0.00

## Significance Statement

Hyponatremia is a major clinical problem with limited therapeutic options. The kidney chloride channel CLC-Ka is an attractive drug target, but its high sequence similarity to CLC-Kb complicates the development of isoform-selective inhibitors, which is required to create a safe drug. Combining cryo-EM structures with molecular dynamics simulations, we define how inhibitors bind at the pore entryway of CLC-Ka and uncover the molecular basis for inhibitor isoform selectivity. We further show that a dynamic extracellular loop can cap the pore and act as a gating element that shapes inhibitor access and engagement. These findings establish a mechanistic foundation for developing improved treatments for hyponatremia.
