# Supplementary material for: Mechanism of Gating and Isoform-Specific Inhibition in Renal CLC Chloride Channels
Source: bioRxiv. 2026 Feb 18:2026.02.17.706469. Preprint. [Version 1] doi: 10.64898/2026.02.17.706469 (PMC12934935; doi:10.64898/2026.02.17.706469)
Supplement: Supplement 1 [file media-1.pdf]

Sequence alignment:

```
                                                                     1
bCLC-K:     1   -----MRVRRGIRGGLDWLKRKLFCVGEDWYFLTVLGVLMALISFTMSFTVGRVVRAHKW   55
hCLC-Ka:   21   LWGPCPHIRRAIQGGLEWLKQKVFRLGEDWYFLMTLGVLMALVSYAMNFAIGCVVRAHQW   80
                         ::**.*:***:***:*:* :******* .*******:*::*.*::* *****:*

56     LYREIGDSHLLRYLSWTVYPVALVSFSSGFSQSITPFSGGSGIPELKTILSGVVLEDYLDIKNFGAKAVG   125
81     LYREIGDSHLLRYLSWTVYPVALVSFSSGFSQSITPSSGGSGIPELKTMLAGVILEDYLDIKNFGAKVVG   150
.      ************************************ ***********:*:**:*************.**
                      2
126    LTCTLASGSTIFLGKVGPFVHLSVMIAAYLGRVRAKATGESENKSKRNEMLVAGAAVGVATVFAAPFSGV   195
151    LSCTLATGSTLFLGKVGPFVHLSVMIAAYLGRVRTTTIGEPENKSKQNEMLVAAAAVGVATVFAAPFSGV   220
.      *:****:***:***********************:.: ** *****:******.****************

196    LFCIEVVSSHFSVWDYWRGFFAATCGAFMFRLLAVFNSEQETITSLYKTSFRVEVPFDLPEIFFFVALGA   265
221    LFSIEVMSSHFSVRDYWRGFFAATCGAFIFRLLAVFNSEQETITSLYKTSFRVDVPFDLPEIFFFVALGG   290
.      **.***:****** ***************:************************:***************.
                                                                       3
266    ICGVASCAYLFCQRKFLGFVKTNPVLSKLMATSKPLYSALAALVLASVTYPPGAGRFMASRLSMREYLDS   335
291    ICGVLSCAYLFCQRTFLSFIKTNRYSSKLLATSKPVYSALATLLLASITYPPGVGHFLASRLSMKQHLDS   360
.      **** *********. **.*:***  ***:*****:*****:*:***:*****.*:*:******:::***

336    LLDHNSWALLTRQASPPWPVEPDPQNLWFEWYHPQFTIFGTLAFFLVMKFWMLILATTIPMPAGYFMPIF   405
361    LFDNHSWALMTQNSSPPWPEELDPQHLWWEWYHPRFTIFGTLAFFLVMKFWMLILATTIPMPAGYFMPIF   430
.      ****:****:*:::******* ***:**:*****:***********************************

406    IFGAAIGRLLGEALSVAFPEGIVAGGVTNPIMPGGYALAGAAAFSGAVTHSISTALLAFELTGQIVHALP   475
431    ILGAAIGRLLGEALAVAFPEGIVTGGVTNPIMPGGYALAGAAAFSGAV-HTISTALLAFELTGQIVHALP   499
.      *:***********:********:************************ *:*******************

476    VLMAVLAANAIAQSCQPSFYDGTIIVKKLPYLPWIRGRKISSHRVTVEHFMNRAITTLAKDTPQEEVVKV   545
500    VLMAVLAANAIAQSCQPSFYDGTIIVKKLPYLPRILGRNIGSHHVRVEHFMNHSITTLAKDTPLEEVVKV   569
.      ********************************* * **:*.**:* ******::********* ******

546    VTSTDMAEYPLVASTESQTLVGTMRRAQLVQALQAEPPSWAPGQQRCLQDILAEGCPVEPVTLKLSPETS   615
570    VTSTDVTEYPLVESTESQILVGIVQRAQLVQALQAEPPSRAPGHQQCLQDILARGCPTEPVTLTLFSETT   639
.      *****::***** ***** *** ::*************:*:*******.***.*****.*  **:****:

616    LHQAHNLFELLNLQSLFVTSQGRAVGFVSWVELEKAISKLTNPPAPKSNSLEVLFQ   671
640    LHQAQNLFKLLNLQSLFVTSRGRAVGCVSWVEMKKAISNLTNPPAPK---------   686
.      ****:***:***********:****** *****::****:********
```

```
Region 1:                   S68→N68
bCLC-K:     TVLGVLMALISFTM-S-FTVGRVVRAHKW
hCLC-Ka:    MTLGVLMALVSYAM-N-FAIGCVVRAHQW

Region 2:                   K165
bCLC-K:     ... LTCTLASGSTIFLG-K-VGPFVHLSVMIA
hCLC-Ka:    ... LSCTLATGSTLFLG-K-VGPFVHLSVMIA

Region 3:                   R355→K355
bCLC-K:     PPGAGRFMASRLSM-R-EYLDSLLDHNSW
hCLC-Ka:    PPGVGHFLASRLSM-K-QHLDSLFDNHSW
```

Figure S1 Sequence alignment in the predicted BIM binding pocket.

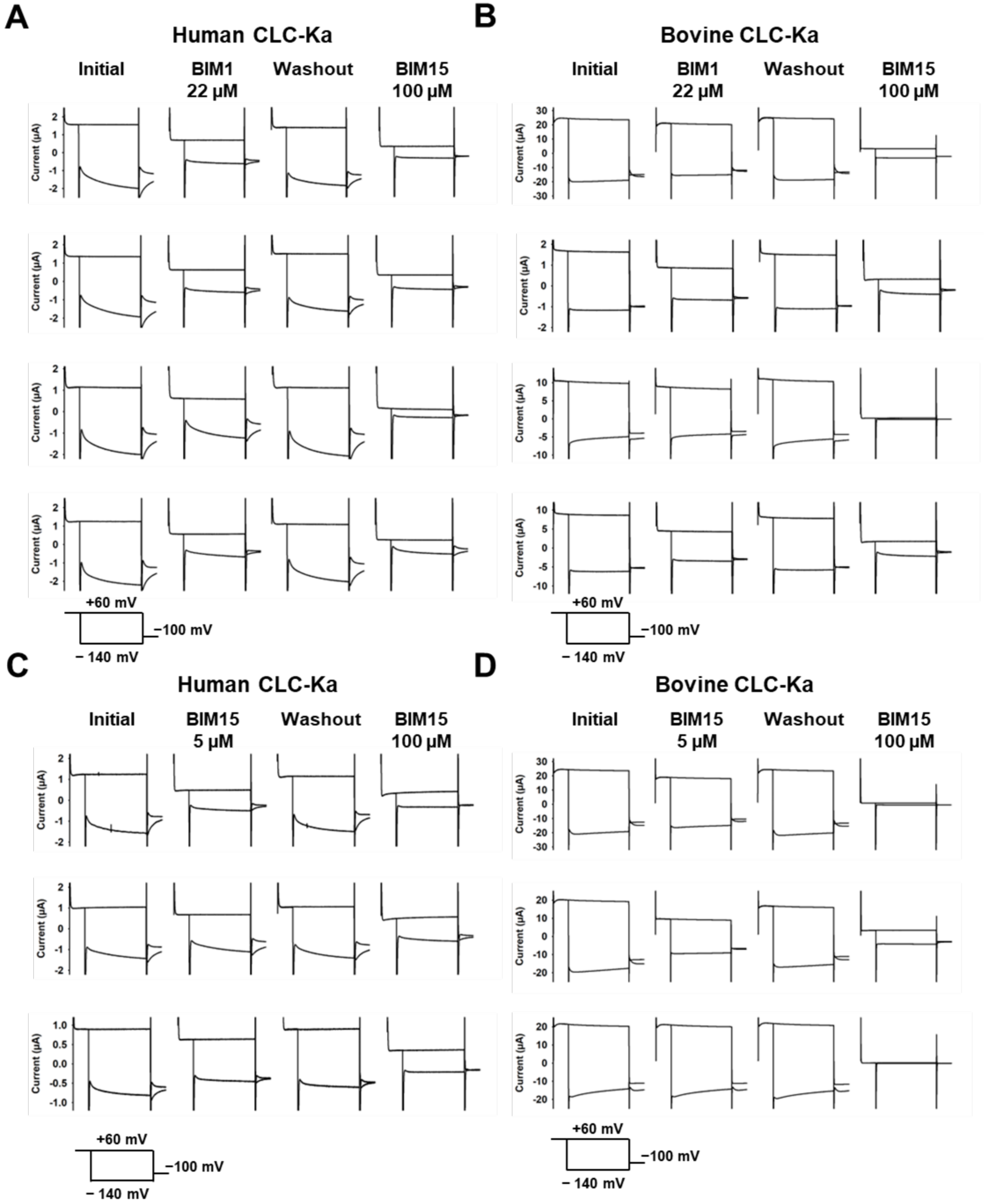

Fig. S2 Primary data for TEVC recordings. Data show recordings from oocytes with overexpressed human CLC-Ka (*A, C*) or bovine CLC-Ka (*B, D*) in the presence of 22 µM BIM1 (*A, B*) or 5 µM BIM15 (*C, D*). Experimental conditions are described in Material and Methods.

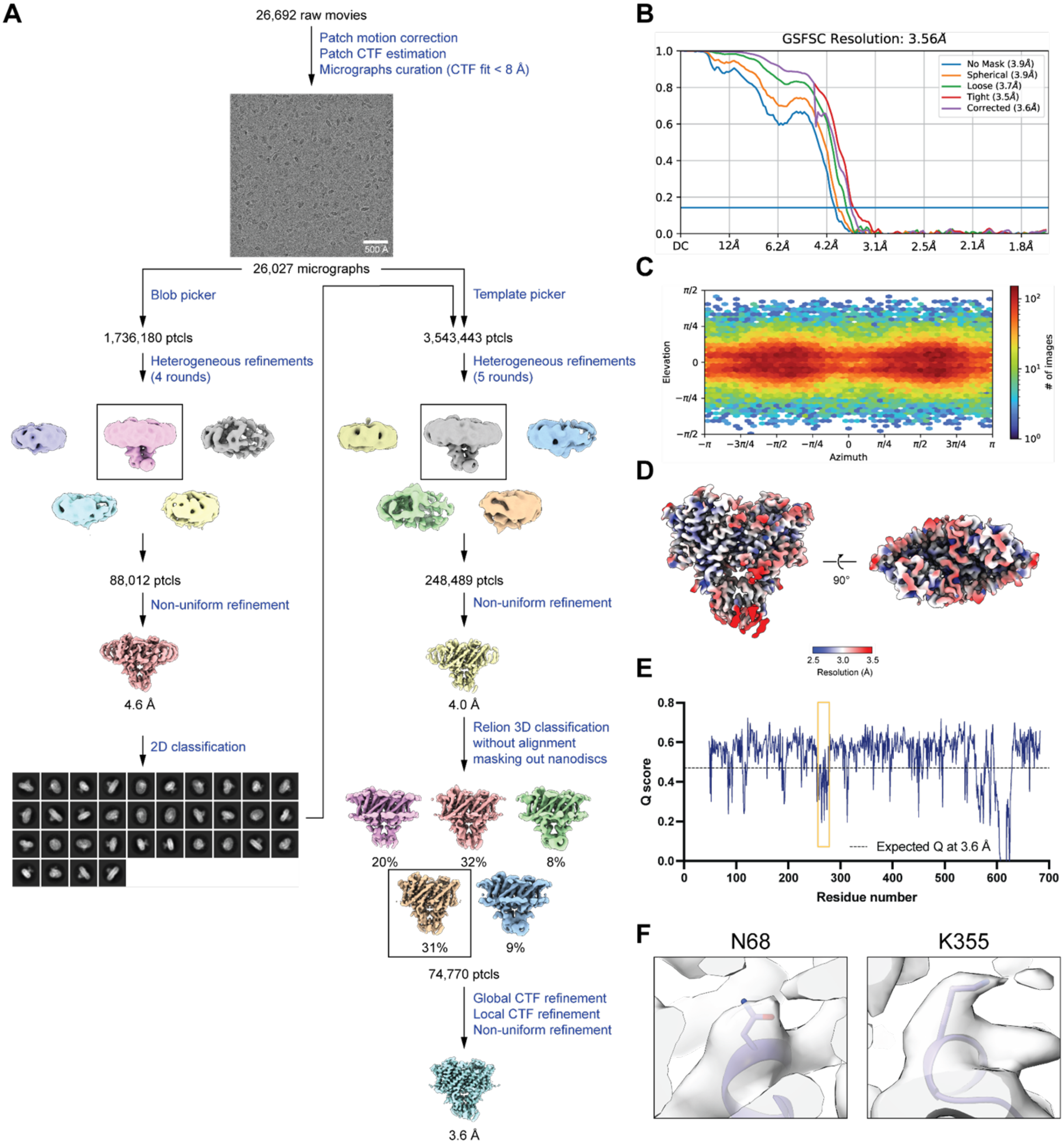

Fig. S3. Cryo EM workflow and validation data for bCLC-Ka. (*A*) Cryo-EM data processing workflow. (*B*) Gold-standard FSC curve. The resolution is estimated based on FSC at 0.143. (*C*) Angular distribution plot. (*D*) Local resolution estimation using Locres in cryoSPARC. (*E*) Per-residue Q-score as a function of residue number. The expected Q-score at the map resolution is indicated by dotted line. The I-J loop region is highlighted by orange box. (*F*) The cryo-EM density and molecular model overlay for residue N68 and K355 (the two residues that were mutated to make bovine CLC-K match human CLC-Ka).

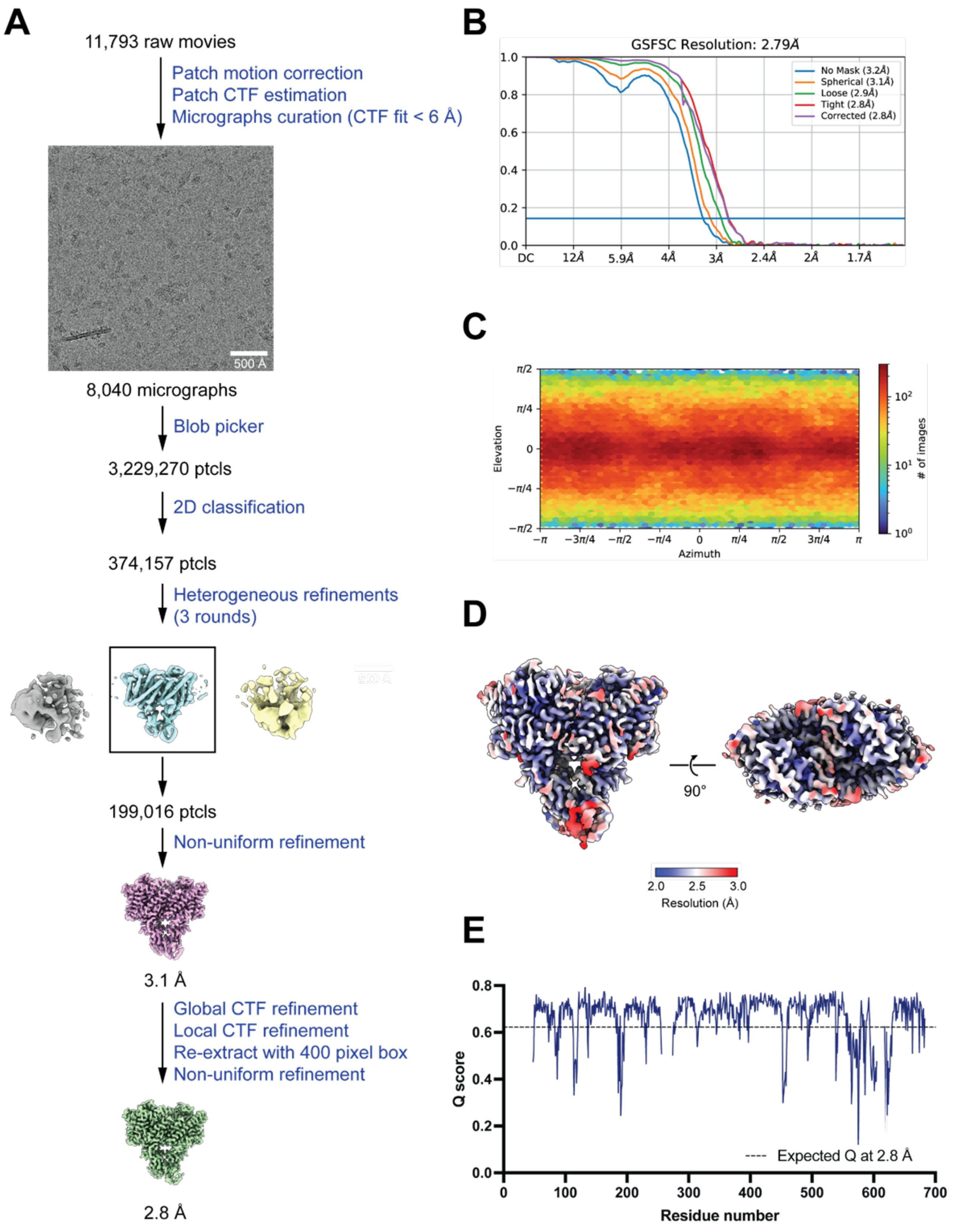

Fig. S4. Cryo EM workflow and validation data for bCLC-Ka with BIM1. (*A*) Cryo-EM data processing workflow. (*B*) Gold-standard FSC curve. The resolution is estimated based on FSC at 0.143. (C) Angular distribution plot. (*D*) Local resolution estimation using Locres in cryoSPARC. (*E*) Per-residue Q-score as a function of residue number. The expected Q-score at the map resolution is indicated by dotted line.

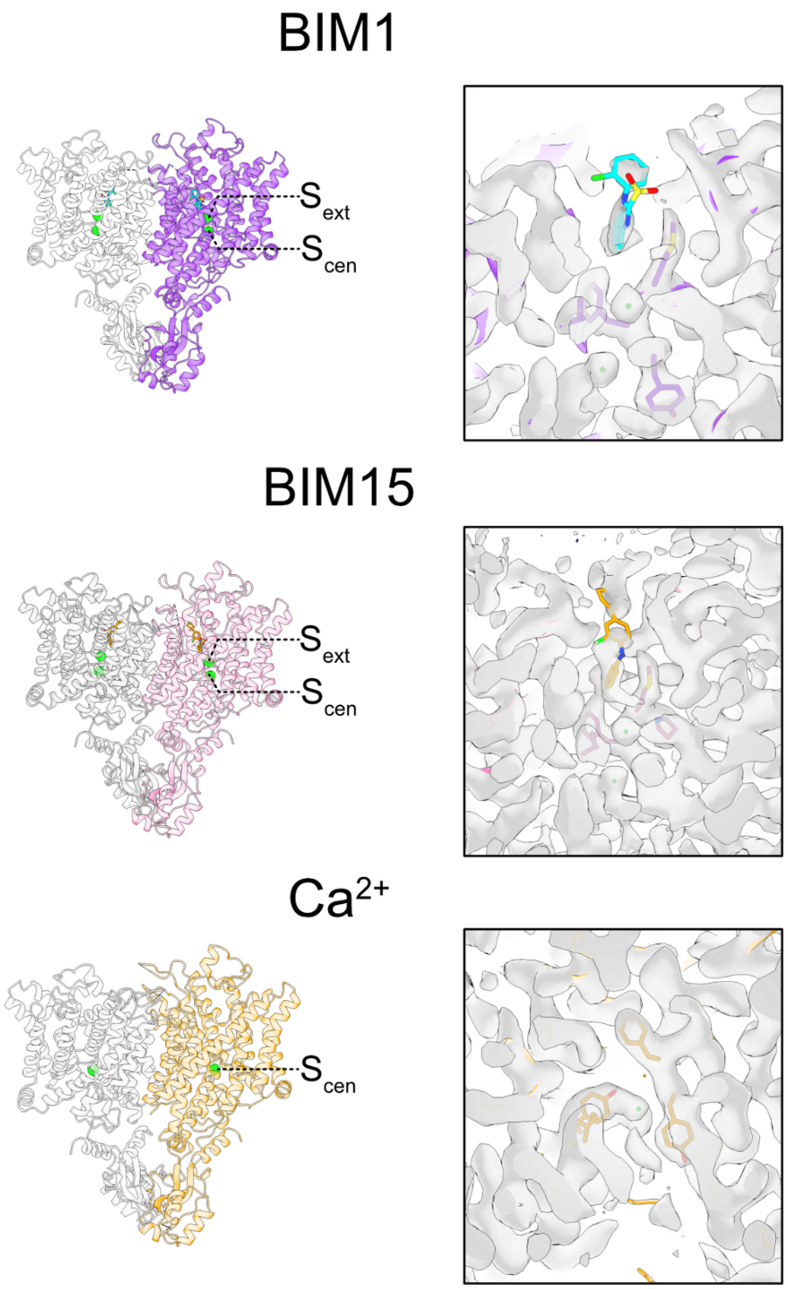

Figure S5. Ion densities for structures determined in this work. The BIM1- and BIM15-bound bCLC-Ka cryo-EM maps display clear densities for Cl^- ions at sites S_{ext} and S_{cen} (maps shown at right, models at left), in contrast to the apo maps (not shown), where Cl^- densities are not well resolved. The Ca^{2+}-bound bCLC-Ka map shows clear density for a Cl^- ion at site S_{cen} (map at right, model at left).

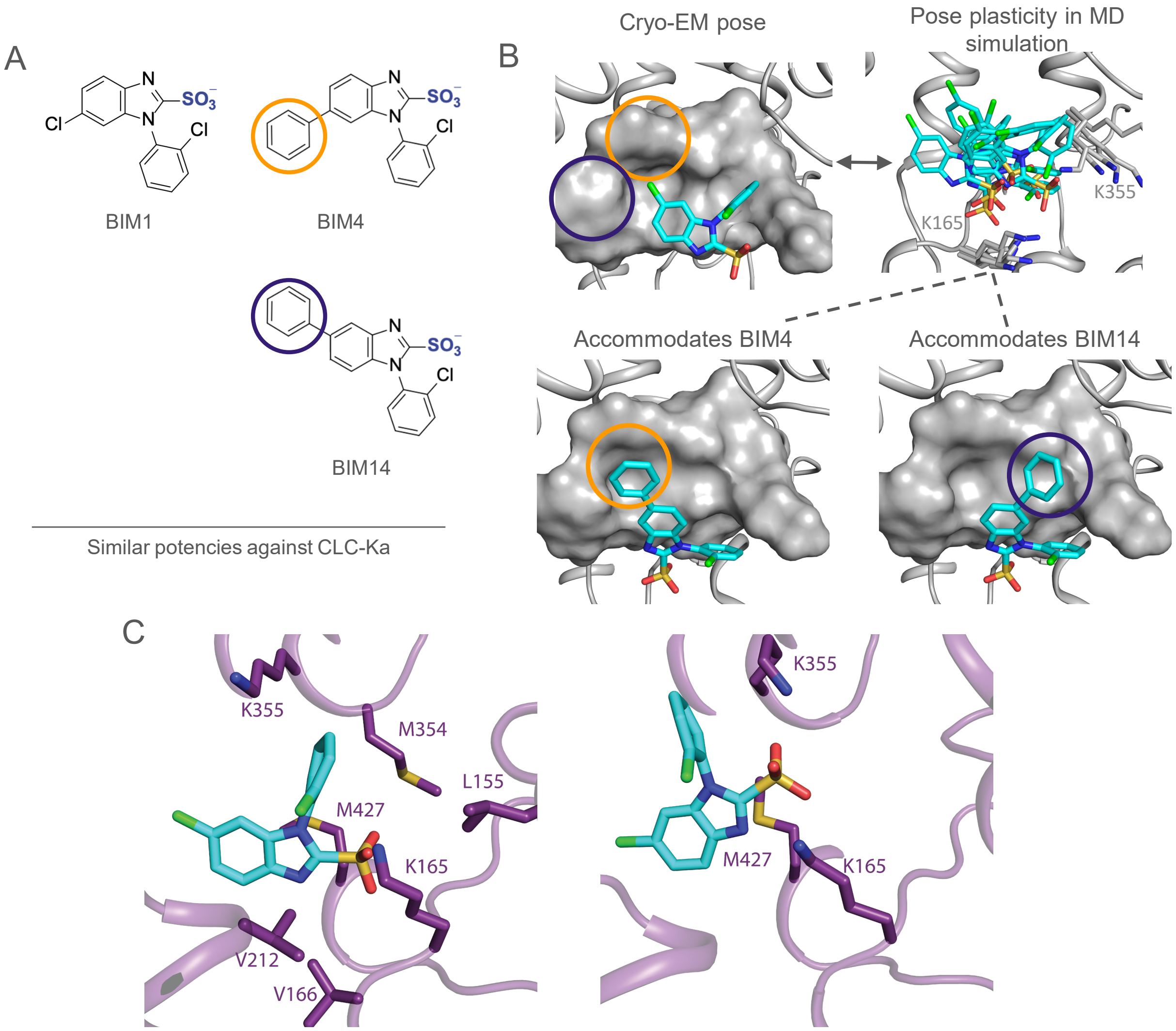

Figure S6 Conformational plasticity of the BIM1 binding pose revealed by molecular dynamics simulations. (*A*) Chemical structures of representative BIM compounds that retain potency but form steric clashes when modeled into the bCLC-Ka structure. BIM4 and BIM14 include additional phenyl groups (highlighted in orange and purple) compared with the parent compound BIM1. (*B*) *Top Left*: Cryo-EM structure of BIM1 bound to bCLC-Ka (gray surface), showing the binding pose (cyan sticks). In this conformation, the additional phenyl groups present in BIM4 and BIM14 would sterically clash with the protein surface (circled). *Top right*: Several frames from MD simulations showing alternative binding poses for BIM1 that could accommodate the additional phenyl groups of BIM4 and BIM14. Residues K165 and K355 are shown as sticks for reference. *Bottom:* Modeled binding poses of BIM4 and BIM14 generated using MD-derived BIM1 poses, illustrating how their phenyl groups could be accommodated in this conformation. (*C*) Representative MD simulation frames showing typical BIM1 interactions with binding pocket residues. All residues within 4Å of BIM1 are shown for each frame. The two poses of BIM1 shown here exhibit different interaction patterns within the binding pocket.

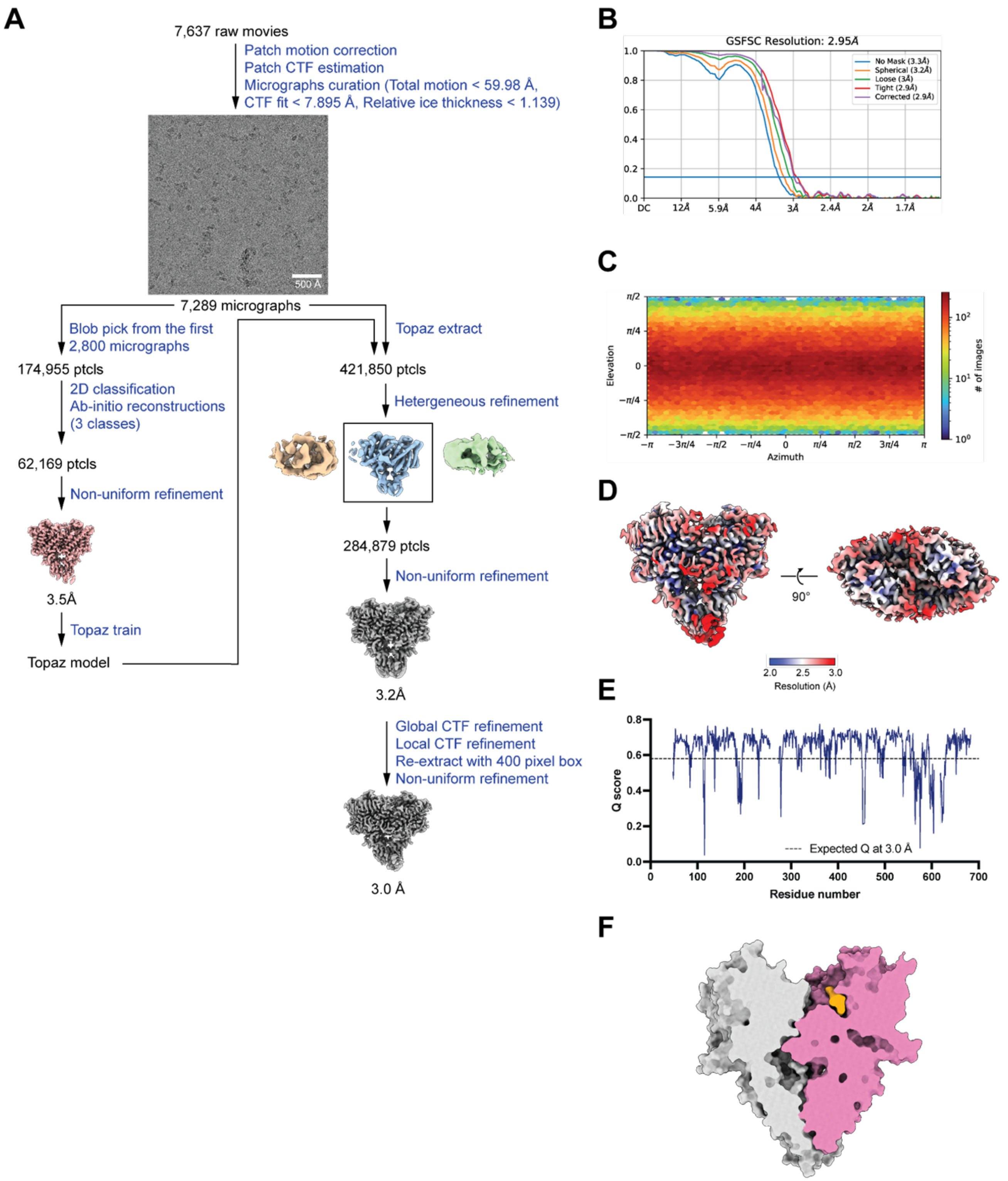

Figure S7. Cryo EM workflow and validation data for bCLC-Ka with BIM15. (*A*) Cryo-EM data processing workflow. (*B*) Gold-standard FSC curve. The resolution is estimated based on FSC at 0.143. (*C*) Angular distribution plot. (*D*) Local resolution estimation using Locres in cryoSPARC. (*E*) Per-residue Q-score as a function of residue number. The expected Q-score at the map resolution is indicated by dotted line. (*F*) Cross-section of the cryo-EM structure of bCLC-Ka with M-BIM15 showing that M-BIM15 directly occludes the chloride pathway.

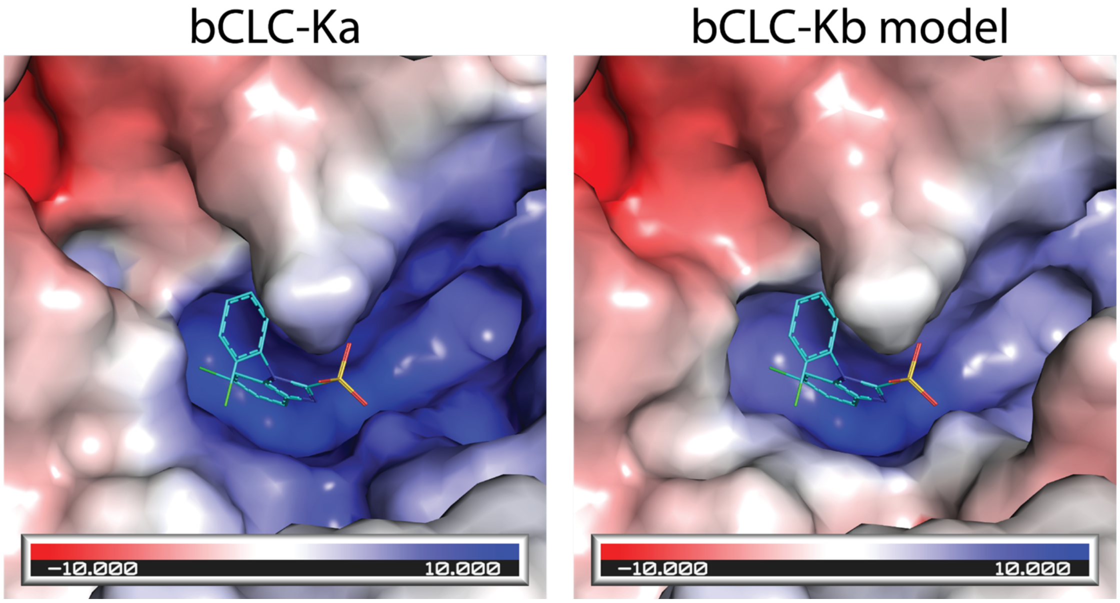

Figure S8. Electrostatic surfaces of bCLC-Ka and a bCLC-Kb model. Electrostatic surface of bCLC-Ka and a bCLC-Kb model, where surface maps are shown on a scale from −10 kT/e (red; negative) to +10 kT/e (blue; positive). BIM1 is shown as cyan lines. These surface maps reveal a more hydrophobic binding pocket in bCLC-Kb versus bCLC-Ka, consistent with previous calculations done on homology models of CLC-Ka and CLC-Kb.

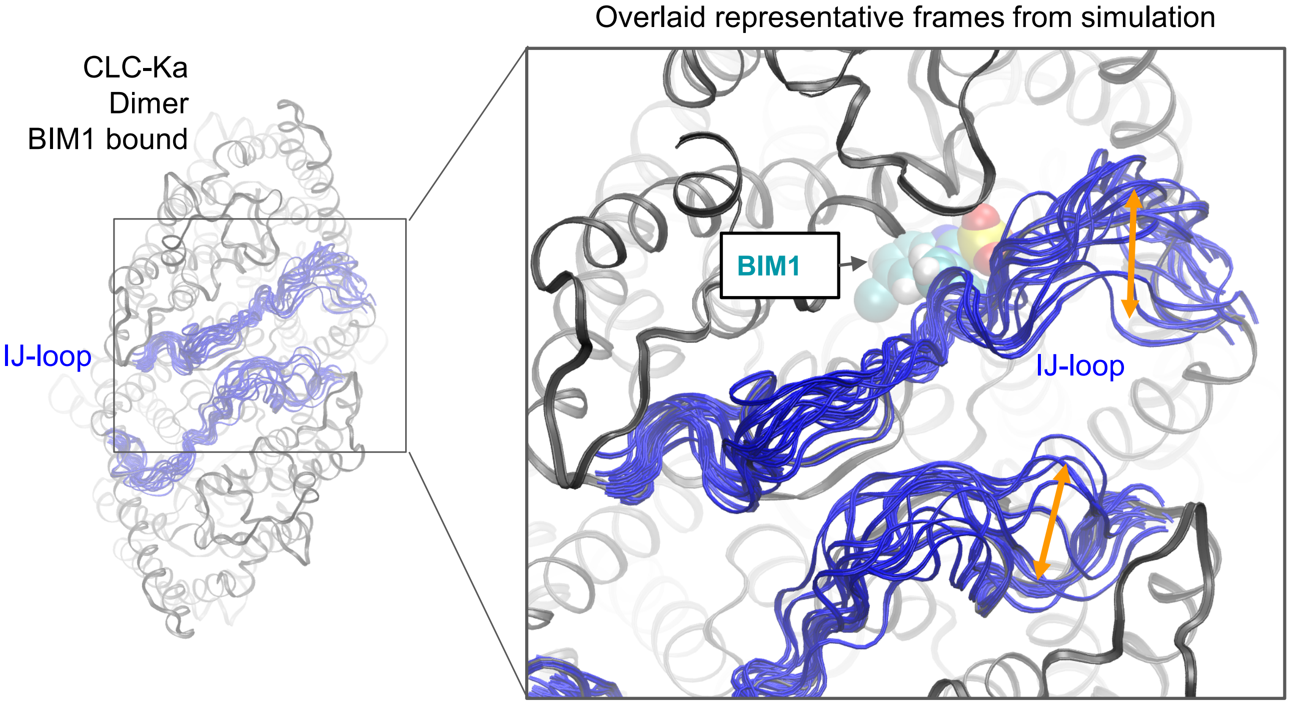

Fig. S9 I-J loop flexibility observed in molecular dynamics simulations. Several representative simulation frames of the I-J loop (one frame per every 100 ns) are superimposed onto the experimentally derived structure of bCLC-Ka bound to BIM1. The I-J loop position for each frame is shown as a blue ribbon, while the bCLC-Ka structure is shown as gray ribbons. The experimentally derived pose for BIM1 is displayed for reference and shown as spheres. The I-J loop shows clear flexibility over the course of the simulations, sampling positions that intermittently block access to the BIM1 site.

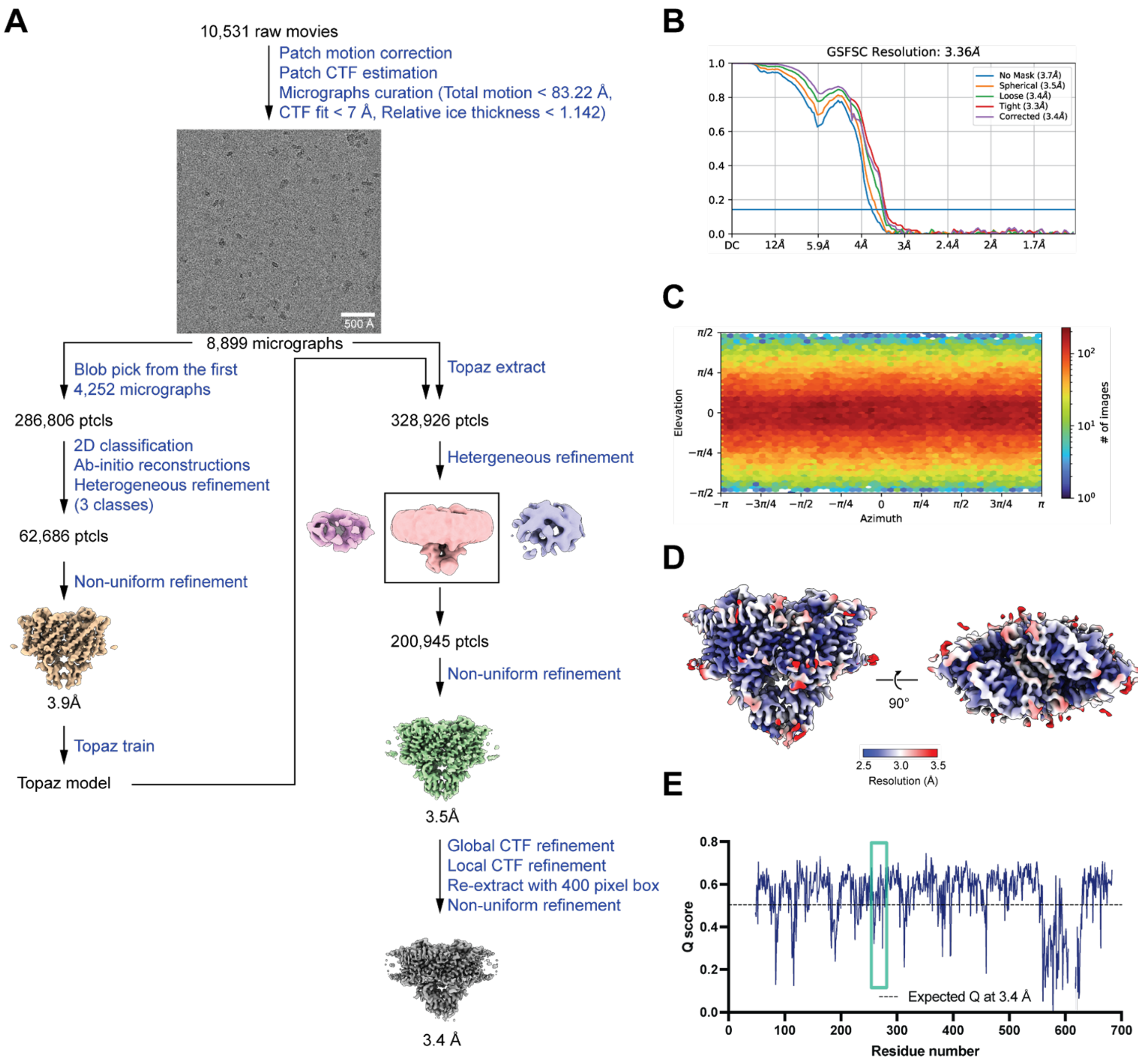

Fig. S10. Cryo-EM workflow and validation data for bCLC-Ka with 100 mM Ca^{2+}. (*A*) Cryo-EM data processing workflow. (*B*) Gold-standard FSC curve. The resolution is estimated based on FSC at 0.143. (*C*) Angular distribution plot. (*D*) Local resolution estimation using Locres in cryoSPARC. (*E*) Per-residue Q-score as a function of residue number. The expected Q-score at the map resolution is indicated by dotted line. The I-J loop region is indicated by a green box.

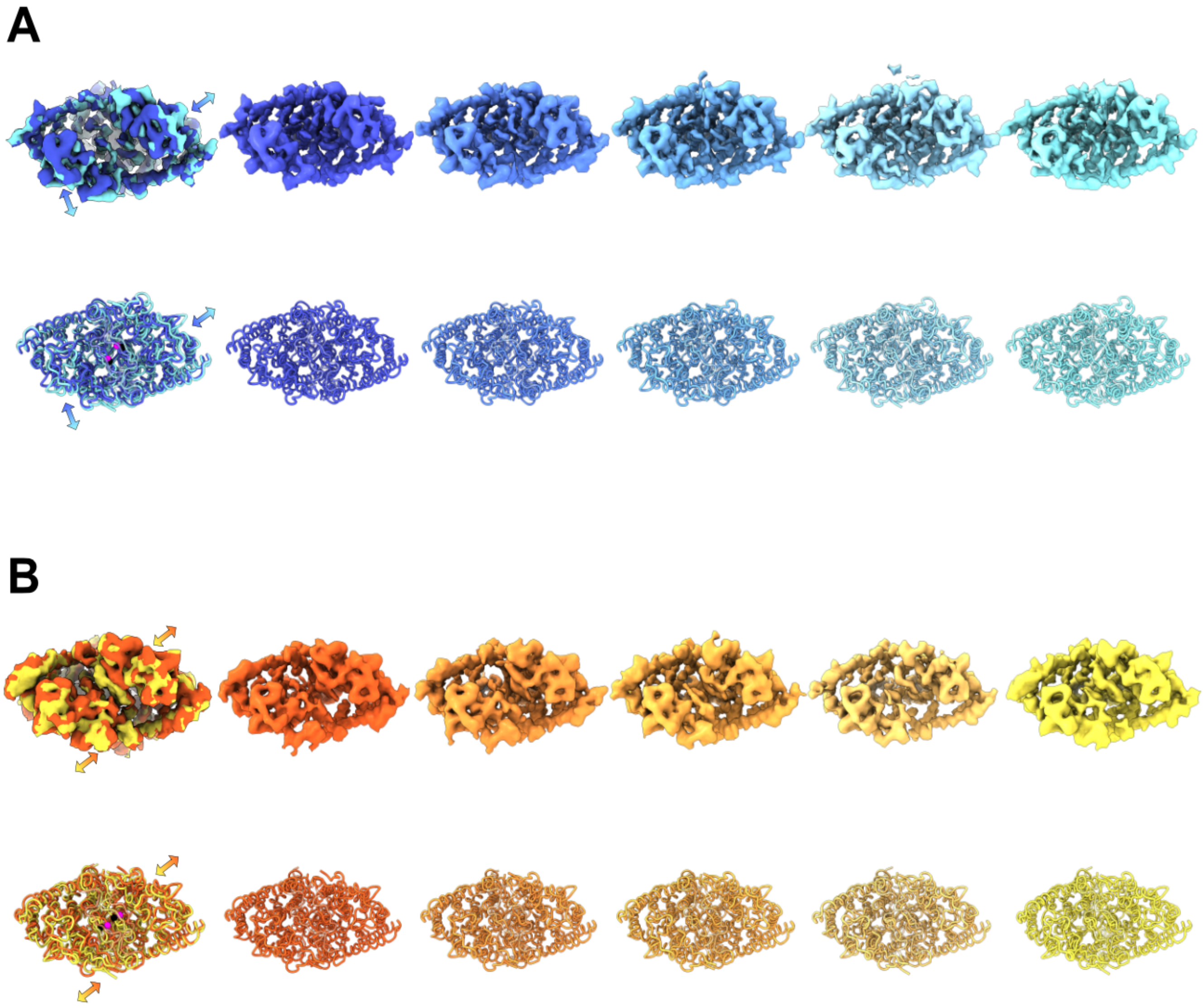

Fig. S11. GMM analysis shows asymmetric subunit motion in apo bCLC-Ka and synchronized, concerted motion in bCLC-Ka with 100 mM Ca^{2+}. Shown here are the detailed GMM-derived motions that underlie the summary presented in Fig. 6. (*A*) *Apo bCLC-Ka.* The top left panel shows overlays of five density maps along the principal motion pathway identified in GMM latent space. The bottom left panel shows the corresponding overlaid structural models, with arrows indicating the direction of motion. The panels to the right display the five individual maps and models without overlays. (*B*) *Ca^{2+}-bound bCLC-Ka.* Panels are arranged as in (*A*).
